# Accuracy assessment of fusion transcript detection via read-mapping and de novo fusion transcript assembly-based methods

**DOI:** 10.1186/s13059-019-1842-9

**Published:** 2019-10-21

**Authors:** Brian J. Haas, Alexander Dobin, Bo Li, Nicolas Stransky, Nathalie Pochet, Aviv Regev

**Affiliations:** 1 Broad Institute of MIT and Harvard, Cambridge, MA 02142 USA; 2 Cold Spring Harbor Laboratory, Cold Spring Harbor, NY 11724 USA; 3 Center for Immunology and Inflammatory Diseases, Division of Rheumatology, Allergy, and Immunology, Massachusetts General Hospital and Harvard Medical School, Boston, MA 02129 USA; 4 Celsius Therapeutics, Cambridge, MA 02139 USA; 5 Ann Romney Center for Neurologic Diseases, Department of Neurology, Brigham and Women’s Hospital, Harvard Medical School, Boston, MA 02115 USA; 6 Howard Hughes Medical Institute, and Koch Institute for Integrative Cancer Research, Department of Biology, Massachusetts Institute of Technology, Cambridge, MA 02140 USA

**Keywords:** Fusion, RNA-seq, Cancer, Benchmarking, STAR-Fusion, TrinityFusion

## Abstract

**Background:**

Accurate fusion transcript detection is essential for comprehensive characterization of cancer transcriptomes. Over the last decade, multiple bioinformatic tools have been developed to predict fusions from RNA-seq, based on either read mapping or de novo fusion transcript assembly.

**Results:**

We benchmark 23 different methods including applications we develop, STAR-Fusion and TrinityFusion, leveraging both simulated and real RNA-seq. Overall, STAR-Fusion, Arriba, and STAR-SEQR are the most accurate and fastest for fusion detection on cancer transcriptomes.

**Conclusion:**

The lower accuracy of de novo assembly-based methods notwithstanding, they are useful for reconstructing fusion isoforms and tumor viruses, both of which are important in cancer research.

## Background

Chromosomal rearrangements leading to the formation of fusion transcripts are a frequent driver in certain cancer types, including leukemia and prostate cancer [1], and contribute to many others [2]. These include BCR–ABL1, found in ~ 95% of chronic myelogenous leukemia (CML) patients [3]; TMPRSS2–ERG in ~ 50% of prostate cancers [4]; and DNAJB1–PRKACA, the hallmark and likely driver of fibrolamellar carcinoma [5]. Determining the driver of a given tumor is important to inform diagnosis and therapeutic strategies. For example, tyrosine kinase inhibitors have been highly effective in the treatment of tumors harboring kinase fusions in leukemia and other cancers [6–9].

Transcriptome sequencing (RNA-seq) has emerged as an effective method to detect fusion transcripts in the precision medicine pipeline. While point mutations and indels can be readily captured from whole exome sequencing (WES), detecting genome rearrangements typically requires whole genome sequencing (WGS). RNA-seq yields the “expressed exome” of the tumor, capturing only the transcriptionally active regions of the genome, and thus provides a cost-effective means to acquire evidence for both mutations and structural rearrangements involving transcribed sequences, which can reflect on functionally relevant changes in the cancer genome.

**Fig. 1:**
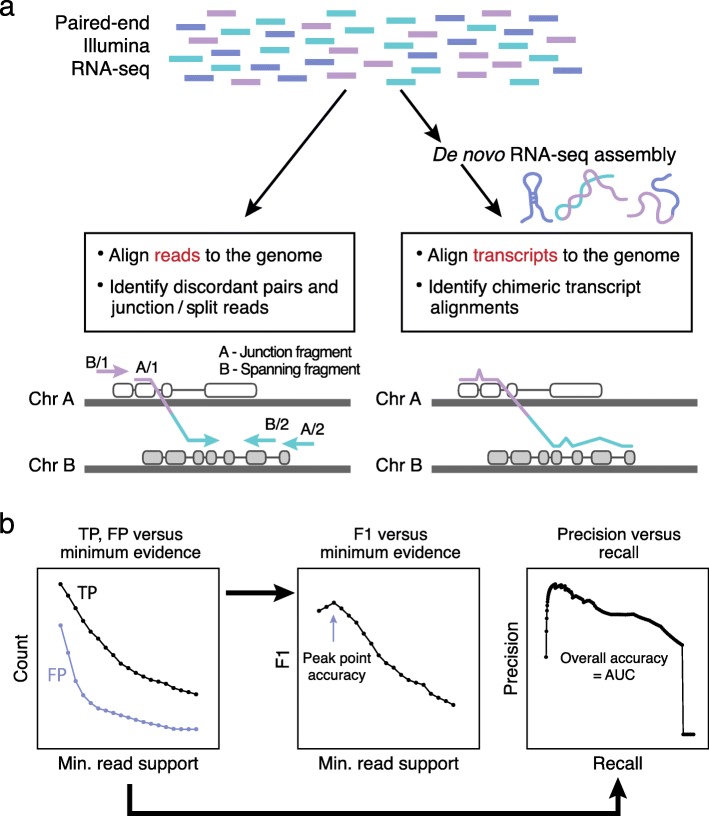
Methods for fusion transcript prediction and accuracy evaluation. **a** The two general paradigms for fusion transcript identification include (left) mapping reads to the genome and capturing discordant read pairs and chimeric read alignments and (right) performing genome-free de novo transcript assembly followed by identification of chimeric transcript alignments. **b** Given a well-defined truth set of fusions, true- and false-positive predictions are tallied according to minimum threshold for fusion-supporting reads. F1 accuracy values are computed at each minimum evidence threshold to determine the threshold that yields peak prediction accuracy for each method. Similarly, precision and recall values are computed at each minimum evidence threshold, plotted as a precision-recall curve, and the area under the curve (AUC) is computed as a measure of overall prediction accuracy

Over the past decade, multiple bioinformatics methods and software tools have been developed to identify candidate fusion transcripts from RNA-seq (reviewed in [10, 11]), with select methods leveraged in recent efforts to build catalogs of fusions across thousands of tumor samples [12, 13]. Following the two general strategies for RNA-seq analyses [14], RNA-seq-based fusion detection falls into two conceptual classes: (1) mapping-first approaches that align RNA-seq reads to genes and genomes to identify discordantly mapping reads that are suggestive of rearrangements and (2) assembly-first approaches that directly assemble reads into longer transcript sequences followed by identification of chimeric transcripts consistent with chromosomal rearrangements (Fig. 1a). Evidence supporting predicted fusions is typically measured by the number of RNA-seq fragments found as chimeric (split or junction) reads that directly overlap the fusion transcript chimeric junction, or as discordant read pairs (bridging read pairs or fusion spanning reads) where each pair of reads maps to opposite sides of the chimeric junction without directly overlapping the chimeric junction itself (Fig. 1a).

Implementations of the various prediction methods vary in the read alignment tools employed, the genome database and gene set resources used, and criteria for reporting candidate fusion transcripts and for filtering out likely false positives. Available fusion predictors vary in prediction accuracy, installation complexity, execution time, robustness, and hardware requirements. Depending on the fusion prediction tool chosen, processing one RNA-seq sample containing tens of millions of reads can take several days’ worth of computing and result in a list of hundreds to thousands of gene fusion candidates, including many likely false positives, with little evidence supporting these predictions. Thus, fast and accurate methods for fusion detection are urgently needed, particularly as RNA-seq is increasingly adopted in precision medicine and clinical diagnostics.

Earlier evaluations of fusion prediction methods based on RNA-seq have highlighted the shortcomings of contemporary methods, but were mostly limited to small numbers of fusion candidates, compared few tools, and relied heavily on simulated test data for accuracy assessment [15, 16]. Here, we advance fusion transcript prediction benchmarking to include thousands of fusion transcripts at varied expression levels, devise a strategy to benchmark fusion accuracy leveraging real cancer transcriptome data without a priori defined gold standard truth sets, and test a large number of tools. Specifically, we evaluate 23 different fusion detection methods (from 19 different software packages) that can leverage solely RNA-seq as input for fusion transcript detection (Table 1, Fig. 1b). This includes two software packages that we developed, STAR-Fusion and TrinityFusion: STAR-Fusion leverages chimeric and discordant read alignments identified by the STAR aligner [18, 51, 52] to predict fusions and TrinityFusion leverages chimeric reads and Trinity de novo transcriptome assembly [48, 53] to reconstruct fusion transcripts and identify fusion candidates. We assess each method, evaluating sensitivity and specificity of fusion detection, and identify those methods best suited for accurate fusion detection from cancer RNA-seq.

**Table 1:** RNA-seq-based fusion transcript predictors evaluated

Method	Class*	Brief overview of methodology
Arriba [17]	R	Arriba extracts gene fusions from the chimeric alignments reported by STAR [18] by applying a collection of filters which recognize frequent types of artifacts found in RNA-Seq data.
ChimeraScan [19]	R	Identifies candidate fusions from discordant Bowtie [20] genome alignments. Unmapped reads are trimmed and realigned. Junction breakpoint reads are resolved by aligning to candidate fused exons. Fusions are filtered based on abundance of fusion-supporting reads.
ChimPipe [21]	R	The GEMtools RNA-seq pipeline [22] and GEM alignment utility [23] are used to capture discordant and chimeric read alignments, and fusion candidates are filtered according to fusion evidence and additional gene-based filters.
deFuse [24]	R	Aligns reads to spliced and unspliced gene sequences using Bowtie [20], resolves split read junctions using a novel dynamic programming algorithm, and uses an AdaBoost classifier to discriminate between likely true vs. false fusions.
EricScript [25]	R	BWA [26] is used to align reads to the genome. Discordant reads are used to identify candidate gene fusions. BLAT [27] is then used in an iterative local alignment step to define precise fusion breakpoints by aligning to customized targets of fused exons. An AdaBoost classifier trained with synthetic data is used to score and rank fusion predictions.
FusionCatcher [28]	R	Leverages a collection of alignment utilities including Bowtie [20], Bowtie2 [29], BLAT [27], and STAR [18] with a collection of customized target databases to identify and characterize fusion candidates. Rigorous filtering of fusion predictions according to gene and fusion annotations is employed.
FusionHunter [30]	R	First uses Bowtie to align reads to the genome and identify candidate fusions based on discordant read pairs. Then creates a “pseudoreference” by positioning candidate fusion genes with canonical ordering, realigns reads using a custom algorithm and identifies both split and spanning reads providing evidence for gene fusions.
InFusion [31]	R	Reads are first aligned to the reference transcriptome using Bowtie2. Unaligned and discordantly aligned reads are further examined in the context of the genome and transcriptome to cluster evidence and define candidate fusions.
JAFFA-Assembly [32]	A	After removing intronic and intergenic region aligning reads defined by Bowtie genome alignments, the remaining reads are assembled using Oases [33] and the assembled contigs are mapped directly to the transcriptome using BLAT. Chimeric BLAT alignments are further assessed as fusion candidates.
JAFFA-Direct [32]	R	After removing intronic and intergenic region aligning reads defined by Bowtie genome alignments, the remaining reads are mapped directly to the transcriptome using BLAT. Chimeric BLAT alignments are further assessed as fusion candidates.
JAFFA-Hybrid [32]	R,A	After removing intronic and intergenic region aligning reads defined by Bowtie genome alignments, the remaining reads are assembled using Oases. Both the assembled transcripts and the original reads that failed to map to the genome are then mapped directly to the transcriptome using BLAT. Chimeric BLAT alignments are further assessed as fusion candidates.
MapSplice [34]	R	An RNA-seq aligner based on Bowtie similar to TopHat [35] and includes fusion-finding capabilities, although specific algorithmic details are lacking.
nFuse [36]	R	Designed for use with WGS-seq and RNA-seq but can be executed with RNA-seq only, leveraging its included deFuse with Bowtie2.
Pizzly [37]	R	Uses a k-mer based strategy to examine reads that do not map to isoforms consistently via kallisto [38] pseudoalignment.
PRADA [39]	R	Reads are aligned to a combined genome and transcriptome reference using BWA. Discordant reads identify fusion candidates, and junction reads are identified by mapping to a database of all possible 5′-3′ chimeric exon junction database.
SOAP-fuse [40]	R	The SOAP2 aligner [41] is used to map reads to genomes and spliced transcripts to identify fusion candidates.
STARChip [42]	R	Uses chimeric reads reported by STAR aimed primarily at identifying circular RNAs but also reports fusion candidates.
STAR-Fusion [43]	R	Uses chimeric read alignments reported by STAR in its Chimeric.out.junction file to identify candidate fusions followed by extensive filtering of likely artifacts.
STAR-SEQR [44]	R	Uses chimeric reads reported by STAR to find fusions.
TopHat-Fusion [45]	R	A modified execution of the TopHat aligner [35, 46] to examine initially unmapped reads as supporting fusion events.
TrinityFusion-C [47]	A	De novo assembles only the chimeric reads defined by STAR using the Trinity assembler [48], and subsequently leverages GMAP [49, 50] for chimera candidate detection.
TrinityFusion-D [47]	A	De novo assembles all input reads using Trinity, and subsequently leverages GMAP for chimera candidate detection.
TrinityFusion-UC [47]	A	De novo assembles both chimeric and unmapped reads defined by STAR using the Trinity assembler, and subsequently leverages GMAP for chimera candidate detection.

*Class of fusion detection method: *R* read mapping, *A* assembly followed by alignment

## Results

### A panel of methods for fusion transcript detection

We assessed 23 methods for fusion transcript detection, including 18 methods primarily based on read-alignments (Table 1): Arriba [17], ChimeraScan [19], ChimPipe [21], deFuse [24], EricScript [25], FusionCatcher [28], FusionHunter [30], InFusion [31], JAFFA-Direct [32], MapSplice [34], nFuse [36], Pizzly [37], PRADA [39], SOAPfuse [40], STARChip [42], STAR-Fusion, STAR-SEQR [44], and TopHat-Fusion [45], and four methods primarily based on transcript assembly: JAFFA-Assembly [32] and three execution modes of TrinityFusion: TrinityFusion-C, TrinityFusion-D, and TrinityFusion-UC. An additional assessed method, JAFFA-Hybrid [32], leverages a combination of both read mapping and de novo assembly approaches. For each method, we used its own recommended alignment and analysis strategy and parameters, as implemented in its respective package (Table 1). We benchmarked each method using simulated data and real RNA-seq from cancer cell lines. In certain cases, we assessed methods in either alternative execution modes, or according to assigned fusion confidence levels (see the “Methods” section). For example, we assessed Arriba using either all predicted fusions or restricting to only those that Arriba labeled as high confidence predictions (Arriba_hc). We assessed TrinityFusion in each of its three alternative execution modes, involving assembly of all input reads (TrinityFusion-D), only chimeric reads (TrinityFusion-C), or both unmapped and chimeric reads (TrinityFusion-UC). We assessed accuracy using both strict and lenient scoring criteria: while strict scoring relied on the pair of gene symbols corresponding to the genes predicted to be fused, lenient scoring also allowed for likely paralogs to serve as acceptable proxies for fused target genes. We show lenient scoring here unless indicated otherwise and point the reader to the “Methods” section for further details and examples.

### Fusion transcript prediction accuracy using simulated data

To assess accuracy in the context of a known ground truth, we applied each of the 23 methods to predict fusions on ten simulated RNA-seq data sets (Additional file 1: Tables S1, S2), each containing 30 M paired-end (PE) reads and each data set incorporating 500 simulated fusion transcripts expressed at a broad range of expression levels. To examine the effect of read length on fusion prediction accuracy, five of the data sets were based on 50 base reads and the other five on 101 base reads, reflecting typical read lengths of contemporary RNA-seq data sets and technologies.

**Fig. 2:**
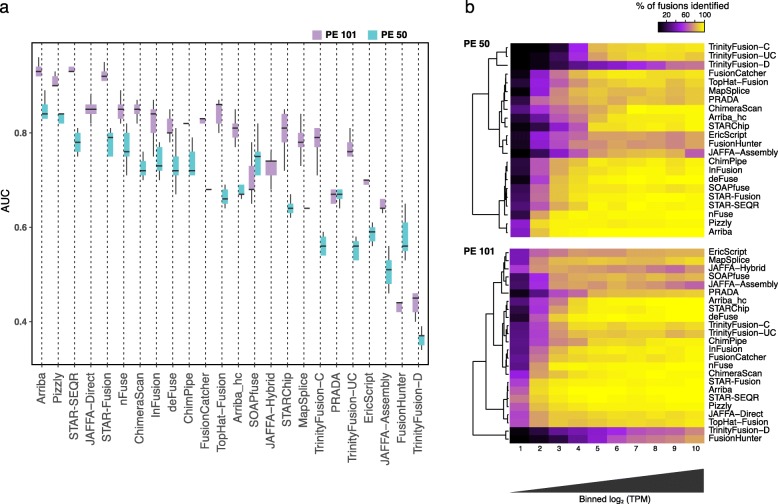
Fusion prediction accuracy on simulated fusion RNA-seq data. **a** Distribution of AUC values across replicates for both the 50 base length (PE 50) and 101 base length (PE 101) simulated paired-end RNA-seq fusion data sets. JAFFA-Hybrid and JAFFA-Direct were incompatible with the shorter PE 50 data set and so only results for longer PE 101 data are shown. **b** Heatmaps illustrating sensitivity for fusion detection according to fusion expression levels. Fusions were divided into bins based on log_2_(TPM) expression levels, and the percent of fusions identified within each expression bin are indicated according to color and intensity

We compared fusion detection accuracy for all methods by several measures (Fig. 1b). We scored true- and false-positive predictions for each method according to minimum fusion evidence support (Additional file 2: Figures S1, S2), and from these, we measured precision (positive predictive value (PPV or P)) and recall (sensitivity or true positive rate (TPR or R)). We calculated the area under the precision-recall (P-R) curve (AUC) as the overall accuracy for each method (Fig. 1b) and examined the distribution of AUC values across samples for each method (Fig. 2a).

### Read length and fusion expression level affect sensitivity for fusion detection

On the simulated data, accuracy was almost entirely a function of sensitivity for fusion detection, as most methods exhibited few false positives (1–2 orders of magnitude lower). Only ChimeraScan accumulated large numbers of false-positive predictions with longer reads, particularly involving fusions predicted with few supporting reads (Additional file 2: Figures S1–S3). Arriba, Pizzly, STAR-SEQR, and STAR-Fusion were the best performers on simulated data, with many close contenders. Methods requiring de novo transcriptome assembly, including TrinityFusion and JAFFA-Assembly, were among the least accurate; each exhibited high precision but suffered from comparably low sensitivity (Additional file 2: Figures S1-S3). Nearly all methods had improved accuracy with longer vs. shorter reads, except for FusionHunter and SOAPfuse, which yielded higher accuracy with the shorter reads, and PRADA, which performed similarly regardless of read lengths examined.

Fusion detection sensitivity was affected by fusion expression level (Fig. 2b). Most methods were more sensitive at detecting moderately and highly expressed fusions, but differed substantially in their ability to detect lowly expressed fusions. These were more readily detected with longer vs. shorter reads, and de novo assembly-based methods made the most notable gains due to increased read length. Of the de novo assembly-based methods, JAFFA-assembly (but not TrinityFusion) had a decrease in sensitivity at the most highly expressed fusions; this could be partly due to JAFFA-assembly using the Oases assembler [33] as opposed to the Trinity assembler [48] used by TrinityFusion. By restricting assembly to chimeric reads or to the combined chimeric and unmapped reads, TrinityFusion-C and TrinityFusion-UC greatly outperformed TrinityFusion-D, which uses all input reads and had low to poor sensitivity for all but the most highly expressed fusions. TrinityFusion-D often preferentially reconstructed the normal (unfused) transcripts instead of rather than in addition to the fusion transcript (e.g., Additional file 2: Figure S4).

### Fusion transcript detection accuracy with RNA-seq from cancer cell lines

We next turned to benchmark fusion detection accuracy using RNA-seq from 60 cancer cell lines. A major challenge in benchmarking using real RNA-seq is that the truth set cannot be perfectly defined. Earlier benchmarking studies of fusion prediction accuracy using RNA-seq from cancer cell lines [15, 28, 32, 54, 55] relied on 53 experimentally validated fusion transcripts from four breast cancer cell lines: BT474, KPL4, MCF7, and SKBR3 [56–59] (Additional file 1: Table S3). However, these fusions arguably represent too small a target truth set for rigorous benchmarking, and the catalog of true fusions for these four cell lines may still be incomplete.

As an alternative, we pursued a “wisdom of crowds” approach [60], where we define true fusions for benchmarking purposes as those predicted by at least *n* different methods, false predictions as those predicted uniquely by any single method, and unsure (unscored) fusions as those non-unique fusions predicted by fewer than *n* different methods (alternative scoring schemes had mostly minimal effects (see the “Methods” section)). To this end, we called fusion predictions on the cancer cell line transcriptomes (Additional file 1: Table S4).

**Fig. 3:**
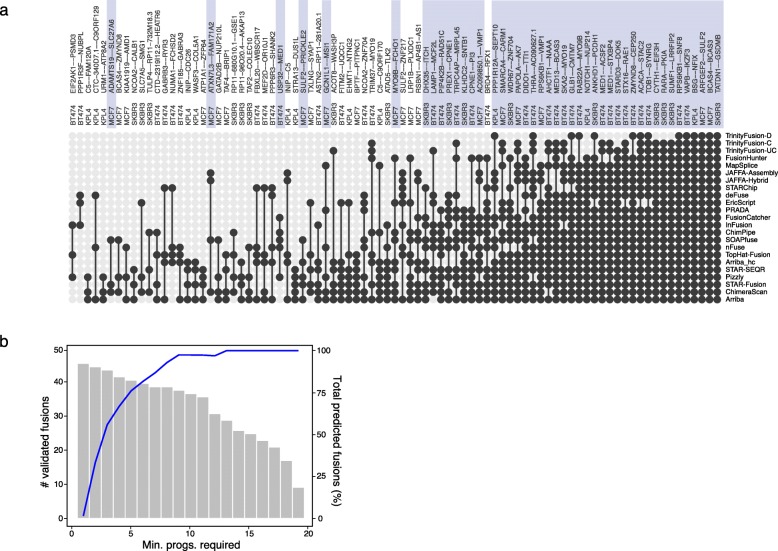
Identification of experimentally validated fusions in breast cancer cell lines BT474, KPL4, MCF7, and SKBR3. **a** All fusions identified by at least three different methods are shown and ranked from being predicted by fewest to most methods in an UpSetR [61] style plot (UpSetR code forked and modified to show individual fusion group memberships here [62]). Previously reported experimentally validated fusions are shaded to facilitate identification. **b** Bar plot showing the number of experimentally validated fusions (left axis) contained within the union of all predictions supported by at least the specified number of fusion prediction methods. Also shown is the corresponding percent of the union of predictions containing experimentally validated fusions (blue line, right axis)

**Fig. 4:**
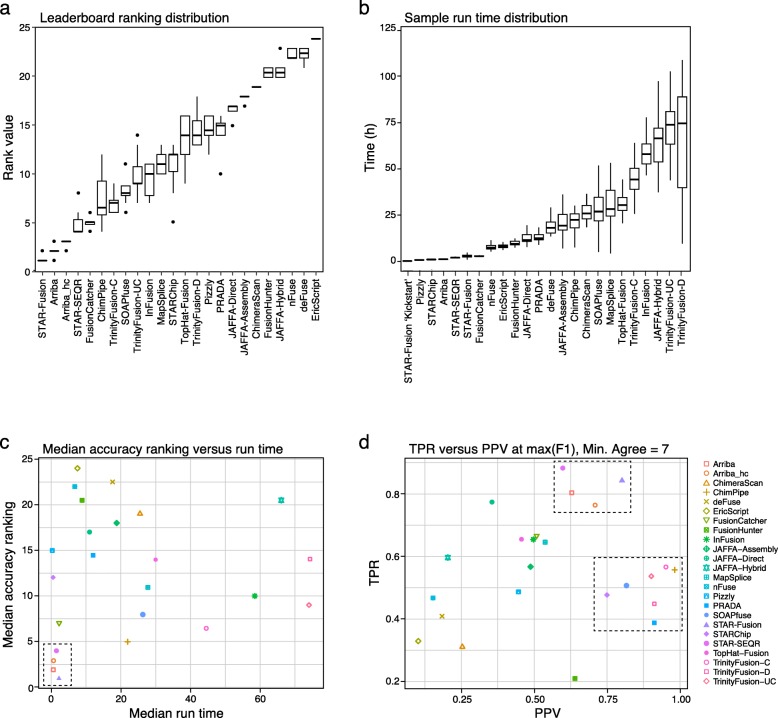
Fusion prediction accuracy on 56 cancer cell lines. **a** The distribution of leaderboard rankings for accuracies assessed using the varied truth sets. Methods are ranked from left to right according to median accuracies. **b** The distributions of execution times for all cancer cell lines are shown. All methods were run on the Broad Institute computing grid with commodity hardware and allocated single cores, with the exception of the two slowest methods, TrinityFusion-UC and TrinityFusion-D, which were each given four cores. **c** Median rankings are plotted vs. median run times, with a black dashed box drawn around the most accurate and fastest methods. **d** The PPV and TPR are shown at maximum point accuracy (F1) for an example trial involving the truth set defined as requiring at least seven methods to agree. The most accurate methods are found to cluster into groups of high sensitivity (top dashed rectangle) or high precision (right dashed rectangle)

To evaluate the merits of this approach, we first composed truth sets this way for the four breast cancer cell lines above. Only one of the 53 experimentally validated fusions (SKBR3|CSE1L--AL035685.1) was predicted by a single method (FusionCatcher). Of a total of 86 fusions predicted by at least three methods, we found 44 experimentally validated fusions (Fig. 3a). As we define potential fusion transcript truth sets by requiring an increasing number *n* of methods to agree, there was an increased enrichment for experimentally validated fusions (Fig. 3b). Thus, by pursuing this approach, rather than being limited to a single truth set, we could explore all possible truth sets defined by a range of values for *n* and examine the distribution of leaderboard rankings for methods across all evaluated truth sets. Accordingly, for the remaining 56 cancer cell line transcriptomes, we evaluated each truth set from *n* = 3 to 10 and examined each method’s leaderboard ranking given each corresponding truth set (Fig. 4a). Notably, relative rankings were mostly stable regardless of which *n* value was used to define the truth set.

### Top-performing fusion prediction methods assessed using cancer RNA-seq

STAR-Fusion had the best ranking across methods in most cases, followed by Arriba and STAR-SEQR (Fig. 4a). Our TrinityFusion-C method was ranked highest among the de novo assembly methods. Notably, the top three ranked methods all leverage the STAR aligner; conversely, STARChip, which also uses STAR, had moderate accuracy, lacking sensitivity and specificity compared to other STAR-based methods. STARChip was primarily developed to detect circular RNAs and so may not have been fully optimized towards the detection of fusions. Restricting Arriba to its self-declared high confidence predictions results in slightly lower accuracy than leveraging its full set of predictions (Fig. 4a, c), stemming from decreased sensitivity that is not sufficiently offset by its increased precision (Fig. 4d).

Execution times varied dramatically across methods (Fig. 4b). The fastest methods include Pizzly, an alignment-free kmer-based approach, followed by the STAR-based methods and FusionCatcher. While STAR-Fusion does not have an alignment-free approach, it does have an “alignment-previous” approach, such that if STAR was run previously as part of another RNA-seq pipeline (e.g., for transcript quantitation), it can use the chimeric junction data file generated during that alignment. This STAR-Fusion “kickstart” mode had the fastest time for fusion discovery (Fig. 4b).

Considering both accuracy and execution time, the most accurate methods, including STAR-Fusion, Arriba, and STAR-SEQR, were also among the fastest (Fig. 4c). ChimPipe and TrinityFusion-C were also found as high ranking for accuracy, but required far longer execution times. Based on sensitivity and precision at a point of peak prediction accuracy, we ascertain two groups of moderate-to-high accuracy predictors, either positioned towards high sensitivity or high precision (Fig. 4d, dashed rectangles). STAR-Fusion, Arriba, and STAR-SEQR comprise the high-sensitivity group, whereas PRADA, ChimPipe, SOAP-fuse, and the different execution modes of TrinityFusion were in the high-specificity group. This pattern was robust for most truth sets explored (Additional file 2: Figure S5).

### Exploration of de novo reconstructed transcripts of potential foreign origin

In addition to de novo reconstruction for fusion transcript identification, TrinityFusion, JAFFA, and other de novo assembly-based methods allow us to explore other transcripts that are not well represented by the reference genome sequence or that are lacking from the reference altogether. In patient samples, these help provide insight into viruses or microbes evident in the sample, which may contribute to tumorigenesis or disease progression [63, 64].

We thus further explored those TrinityFusion-reconstructed transcripts from cancer cell lines that may be of viral or microbial origin. We searched all TrinityFusion (modes D, UC, and C)-reconstructed transcripts against available viral and bacterial sequences using Centrifuge [65] (Additional file 1: Table S5). Most cell lines (56/60) had at least one Trinity-reconstructed transcript classified by Centrifuge as likely of foreign origin. The vast majority of those (77%) were derived from TrinityFusion-UC, followed by TrinityFusion-D (23%), and included only two transcripts from TrinityFusion-C. Next, we aligned all TrinityFusion-UC-reconstructed transcripts against the combined viral, bacterial, and human genome database (blastn [66], *e* value threshold 10^−10^, with reconstructed sequences and alignment coordinates provided in Additional file 1: Table S6). We only detected significant alignments to known mammalian viruses and bacteriophage. Half of the cell lines had evidence of murine type C retrovirus (30/60), and Trinity fully or nearly fully reconstructed these viruses in cell lines VCaP_85 (9.5 kb contig) and G28050.KMM-1.1 (8.4 kb contig), with this pair of viruses sharing 94% nucleotide identity. Consistently, the VCaP_85 retrovirus sequence was previously reported as a xenotropic murine leukemia virus in prostate cancer cell line VCaP [67]. Nine cell lines had evidence of “squirrel monkey retrovirus”—originally identified in a lymphoblastoid cell [68]. Other notable examples included a 40-kb contig corresponding to the phage lambda genome that we reconstructed from the K562 cell line RNA-seq reads (possibly a spike-in control or sequencing library contamination), and a bovine polyomavirus in breast cancer cell line SKBR3, likely reflecting contamination through fetal bovine serum albumin [69].

## Discussion

Fast and accurate fusion detection is important in both cancer research and the precision medicine pipeline. Despite dozens of tools and utilities being available, users have few guidelines as to which to pursue, and developers do not have clear benchmarks to assess the utility of newly proposed methods. Our comprehensive benchmarking shows that only a few of the available tools were both fast and accurate, highlighting those best suited to meet the demands of large-scale tumor sample screening.

In particular, STAR-Fusion, Arriba, and STAR-SEQR had the best combination of speed and accuracy compared to 19 other methods evaluated on cancer transcriptome data. These were also among the most accurate methods when evaluated with simulated RNA-seq, which mostly differentiated methods based on sensitivity rather than precision of fusion prediction. Although FusionCatcher was not among the top performing methods on simulated data, its overall accuracy and execution times were impressive. Note that an earlier version of FusionCatcher (v0994e) had a slightly improved accuracy in our evaluations compared to the contemporary release (v1.10), with the latest release seemingly tuned for improved recall at the cost of reduced precision (Additional file 2: Figures S1–S3, S5). In contrast to an earlier assessment of fusion prediction accuracy that found EricScript to be top-ranking [16], in our assessment, EricScript was the least accurate method on cancer cell line RNA-seq, suffering mostly from a high rate of false positives. Unless indicated otherwise, we used default parameters for all methods. Adjustments in parameters could certainly impact accuracy characteristics, and the framework for benchmarking fusion detection methods that we established here facilitates exploration of the parameter space for further optimization, and exploring accuracy characteristics across software versions.

While our RNA-seq simulations were useful for defining an unambiguous truth set, and evaluating the impact of fusion expression levels and read length, some characteristics of real RNA-seq data are not presently modeled, such as reverse transcription artifacts and off-target transcription (e.g., non-spliced introns and intergenic transcription). Future developments that improve on RNA-seq simulations should further the use of simulated data for benchmarking fusion detection and related methods.

Our application of the “wisdom of crowds” approach towards defining truth sets for benchmarking fusion detection with real cancer transcriptome data allowed us to extend our benchmarking with real data well beyond the small set of cancer cell lines for which there are known experimentally validated fusions. The wisdom of crowds approach enriched for validated fusions when applied to the four breast cancer cell lines. In addition to the 44 validated fusions predicted by at least three methods on the four breast cancer cell lines, we identified additional well-supported fusions that have not yet been experimentally tested to our knowledge. One of these fusions, SULF2--ZNF217 predicted by nine different methods in cell line MCF7, was recently predicted to be a potential driver of breast cancer [70]. Future follow-up investigations are likely to capture experimental evidence for many of these additional fusions as well.

The read-mapping-based approaches to fusion detection have a level of sensitivity that is not met by de novo assembly-based methods, requiring at minimum a small number of fusion-supporting reads that would unlikely assemble into a sufficiently long transcript contig. By restricting the assembly to the chimeric and unmapped reads, TrinityFusion demonstrated greater accuracy in fusion detection than from assembling all of the input reads, presumably due to the reduced search space and the depletion of reads from the non-fused counterparts that could interfere with robust fusion isoform assembly. De novo methods do have other advantages in addition to recovering fusion isoform sequences. By exploring the de novo reconstructed transcripts derived from chimeric and unmapped reads, we identified transcripts of likely foreign origin among many of the cancer cell lines, including tumor viruses. Our TrinityFusion-UC method for assembly and analysis of de novo reconstructed transcripts based on unmapped and chimeric reads should continue to prove useful in future studies that extend to patient samples.

While there have been substantial advances in fusion detection algorithms over the last decade, there remain opportunities for improving fusion transcript prediction accuracy, developing more realistic RNA-seq simulations, and expanding the catalog of experimentally validated fusion transcripts. As sequencing technologies and experimental protocols continue to evolve, the universe of available methods and software will surely continue to expand. Our fusion transcript benchmarking framework provides a flexible system for evaluating these newly developed methods as they become available. All benchmarking software and data are freely available at https://github.com/fusiontranscripts/FusionBenchmarking .

## Conclusion

In applying our fusion transcript benchmarking framework to 21 different methods, leveraging simulated and real cancer RNA-seq, we identified STAR-Fusion, Arriba, and STAR-SEQR as top performers and likely best suited for current applications in processing cancer transcriptome samples. While the de novo assembly-based fusion detection methods are unable to attain the sensitivity of the read-mapping-based approaches, they provide more complete fusion isoform sequence evidence and can reconstruct foreign transcripts such as tumor viruses. Our fusion transcript benchmarking methods and software provide a framework and metrics for systematic benchmarking and evaluation of additional methods as they become available.

## Methods

### Benchmarking fusion prediction accuracy

We assessed fusion prediction accuracy using simulated and real RNA-seq and compared 23 fusion prediction methods including methods we developed and described here: STAR-Fusion [43] and three execution modes of TrinityFusion [47]. Specifically, we downloaded and installed each of (1) Arriba [17], (2) ChimeraScan [19], (3) ChimPipe [21], (4) deFuse [24], (5) EricScript [25], (6) FusionCatcher [28], (7) FusionHunter [30], (8) InFusion [31], (9) JAFFA-Assembly [32], (10) Jaffa-Direct [32], (11) JAFFA-Hybrid [32], (12) MapSplice [34], (13) nFuse [36], (14) Pizzly [37], (15) PRADA [39], (16) SOAPfuse [40], (17) STARChip [42], (18) STAR-SEQR [44], and (19) TopHat-Fusion [45, 46]. To ensure consistency, we reconfigured SOAPfuse and TopHat-Fusion to leverage the GENCODE v19 annotation. Programs and parameters used are provided in Additional file 1: Table S7. Benchmarking data, scripts, and the analysis protocols followed are further provided at [71].

### Simulated fusion transcripts and RNA-Seq

We generated simulated chimeric transcripts using custom scripts, developed and released here as the FusionSimulator Toolkit [72]. FusionSimulator selects two protein-coding genes at random from the GENCODE v19 annotations [73]. It then constructs a fusion transcript by randomly fusing a pair of exons selected at random from each gene, requiring that each gene contributes at least 100 bases of transcript sequence to the generated fusion and that the fusion breakpoint occurs between two exons that have consensus dinucleotide splice sites. In generating a set of fusion genes, any gene participating as a fusion partner is allowed to exist in only one fusion pair.

We simulated RNA-Seq reads using “rsem-simulate-reads” in the RSEM software [74]. RSEM was first used to estimate the expression values of the GENCODE v19 reference transcripts supplemented with the simulated fusion transcripts. Next, the expression values of the simulated fusion transcripts were reset randomly according to a log_2_ distribution of transcripts per million (TPM) expression values in the dynamic range of 1 to 15. Simulated read lengths and read quality characteristics were modeled based on real RNA-seq data sets as described below. Note, however, that while the read sequence simulations model sequence and fragment length characteristics of real RNA-seq data, the current simulations do not model reverse transcription template switching or other important confounding characteristics of real RNA-seq data that are relevant to fusion detection. After directly setting fusion transcript expression values, all transcript expression values were renormalized to TPM values (summing to 1 million) and subject to RNA-seq read simulation using rsem-simulate-reads.

This process was applied separately for ten samples, each generating 500 random fusions and simulating 30 million PE Illumina RNA-seq reads. Half of the simulated samples generated 50 base reads (PE-50) and the other half 101 base reads (PE-101). The PE-50 reads were modeled on short RNA-seq reads generated by the Illumina Human Body Map 2.0 study (ArrayExpress study E-MTAB-513 [75];), and the PE-101 based on a set of cancer cell lines from the Cancer Cell Line Encyclopedia (CCLE) [76] (sources for the targeted data sets are listed in Additional file 1: Table S8). Simulated fusion transcripts and simulated RNA-seq are made available at [77].

### Fusion prediction in cancer cell line transcriptomes

Paired-end Illumina RNA-seq were obtained from 60 publicly available cancer cell line data sets, spanning a variety of cancer types (data sources and representative cancer types are listed in Additional file 1: Table S9). Cancer cell lines included 52 from the CCLE project and further supplemented with 8 other cancer cell lines popularly studied for fusion detection including the breast cancer cell lines BT474, KPL4, MCF7, and SKBR3 [56]; VCaP (prostate cancer); LC2/ad and H2228 (lung adenocarcinoma); and K562 (erythroleukemia). To facilitate benchmarking and runtime analysis, 20 million paired-end reads were randomly sampled from each data set and targeted for fusion prediction. All sampled cancer cell line RNA-seq data targeted for fusion discovery are available at [78]. For CCLE RNA-seq, the names of the reads leveraged are provided, and the sequences must be obtained from the CCLE project according to their data use agreement. For other publicly available cell line RNA-seq, the FASTQ files as used here are directly accessible.

### Fusion prediction accuracy computation

True-positive (TP), false-positive (FP), and false-negative (FN) fusion predictions were assessed for each method. The true positive rate (TPR; or recall or sensitivity), positive predictive value (PPV, precision), and F1 accuracy measure (the harmonic mean of TPR and PPV) were computed according to standards:

$$ \mathrm{Recall}=\mathrm{TP}\mathrm{R}=\mathrm{TP}/\left(\mathrm{TP}+\mathrm{FN}\right) $$

$$ \mathrm{Precision}=\mathrm{PPV}=\mathrm{TP}/\left(\mathrm{TP}+\mathrm{FP}\right) $$

$$ \mathrm{F}1=2\ast \left(\mathrm{TPR}\ast \mathrm{PPV}\right)/\left(\mathrm{TPR}+\mathrm{PPV}\right) $$

TP and FP were assessed at each minimum supporting evidence threshold to generate precision-recall curves, and prediction accuracy was measured as the area under the precision-recall curve (AUC), which is better suited than the popular receiver operating characteristic curve for studies such as fusion prediction where the numbers of true negatives (at least ~ 20k^2^, considering possible gene pairings) far exceed the number of true-positive fusions [79].

Fusion accuracy computations as described here were performed using lenient scoring criteria as follows. Given a true fusion pair “GeneA–GeneB”, the following predictions would be scored as true positives:

- “GeneB–GeneA” having the fusion partners in reverse order
- “GeneZ–GeneB” where GeneZ physically overlaps the genomic coordinates of GeneA
- “GeneZ–GeneB” where GeneZ is a potential paralog of GeneA

Further, in the case where multiple fusions are predicted and there is uncertainty as to which paralogous family member is the true fusion partner (i.e., “GeneA–GeneB” is predicted in addition to GeneZ–GeneB, the fusion GeneA–GeneB is scored as a single TP, and GeneZ–GeneB is ignored. Each of the rules described above applies identically for cases where GeneZ replaces GeneB instead of GeneA. This lenient scoring mostly serves to reduce numbers of FP resulting from paralog confusion or uncertainty, as shown in Additional file 2: Figure S6.

For the cancer cell lines, truth sets were defined by fusions agreed upon by at least *n* different methods. The pairwise correlations among fusion predictions by methods are shown in cr 2: Additional file 2: Figure S7. To avoid including highly correlated methods that would otherwise bias the wisdom of crowds approach, JAFFA-Hybrid was excluded due to its high correlation with JAFFA-Direct. Furthermore, TrinityFusion-C but not the other TrinityFusion modes contributed votes. Since we did not utilize DNA-seq data here, nFuse was executed using its included version of deFuse as instructed [80]; since nFuse (deFuse) was not found highly correlated with the original deFuse predictions, we retained both. Finally, while Arriba_hc was scored separately from Arriba, those fusion predictions did not contribute votes independently from Arriba. Fusions predicted by at least two methods but fewer than *n* methods were treated as uncertain and ignored. Uniquely predicted fusions (those not predicted by at least two of the counted methods) were assigned as FP. The effect of using alternative scoring schemes that penalize the uncertain predictions or fail to account for paralog uncertainty are shown in Additional file 2: Figures S8,S9.

### Fusion prediction by STAR-Fusion

STAR-Fusion is a component of the Trinity Cancer Transcriptome Analysis Toolkit (CTAT) project [81] and leverages a precompiled bundle of genomic resources and metadata provided as a CTAT genome library (described below). The STAR-Fusion pipeline (Additional file 2: Figure S10) takes Illumina RNA-seq data as input and generates lists of candidate fusion transcripts as output. STAR-Fusion release v1.5 was used with the STAR aligner v2.6.1a. The STAR aligner command is as follows (example provided for cell line K562 test data):

STAR –genomeDir CTAT_GENOME_LIB/GRCh37_gencode_v19_CTAT_lib_Feb092018/ctat_genome_lib_build_dir/ref_genome.fa.star.idx --outReadsUnmapped None --chimSegmentMin 12 --chimJunctionOverhangMin 12 --chimOutJunctionFormat 1 --alignSJDBoverhangMin 10 --alignMatesGapMax 100000 --alignIntronMax 100000 --alignSJstitchMismatchNmax 5 -1 5 5 --runThreadN 1 --outSAMstrandField intronMotif --outSAMunmapped Within --outSAMtype BAM Unsorted --readFilesIn K562/reads. SRR521460_1.fastq.20 M.fq.gz K562/reads. SRR521460_2.fastq.20 M.fq.gz--outSAMattrRGline ID:GRPundef --chimMultimapScoreRange 10 --chimMultimapNmax 10 --chimNonchimScoreDropMin 10 –peOverlapNbasesM 0.1 --genomeLoad NoSharedMemory --twopassMode Basic --readFilesCommand “gunzip -c”.

The resulting “Chimeric.out.junction” file containing all chimeric split and discordant reads is leveraged as input to STAR-Fusion.

STAR-Fusion maps the reads to exons of reference gene structure annotations based on coordinate overlaps. STAR-Fusion primarily focuses on filtering the alignment evidence and preliminary fusion predictions to remove likely artifacts and likely false-positive predictions. First, read alignments between pairs of genes that are localized to sequence similar regions between those genes are excluded (Additional file 2: Figure S11). A database of all-vs-all blastn matches between all reference cDNA sequences is queried to identify regions of sequence similarity between candidate fusion genes. If chimeric read alignment evidence overlaps sequence similar regions, the alignment is discarded. Duplicate paired-end read alignments are removed, and the remaining alignments are assigned to preliminary fusion gene pair candidates. STAR-Fusion selects those candidate gene pairs for which the fusion-supporting evidence indicates a sense-sense orientation between the fusion pairs and scores them according to the number of split reads supporting the fusion breakpoint and the number of paired-end fragments that span the breakpoint.

These preliminary fusion gene candidates are filtered in two stages: a basic filtering stage that requires minimum fusion evidence support and an advanced filtering stage that examines characteristics of the genes involved in the candidate fused gene pairs. The basic filtering requires that at least two RNA-seq fragments support the fusion and at least one of the reads is a split read that defines the fusion breakpoint within the spliced transcripts (Additional file 2: Figure S12a). If the fusion breakpoint does not correspond to annotated reference exon splice sites, then at least three split reads are required to provide evidence for that breakpoint. If there are no spanning fragments and only split reads supporting the fusion, then we require at least 25 base length alignment on each side of the splice junction (Additional file 2: Figures S12b).

The advanced fusion filtering involves a series of operations that examine characteristics of the fusion genes in the context of the individual fusion pair and in comparison to other fusion predictions called in that sample:

- Fusion paralog filter: excludes fusion candidate GeneA–GeneB if GeneA is a likely paralog of GeneB. Also, if there exists a candidate “GeneA–GeneC” such that GeneC is a likely paralog of GeneB, and the fusion evidence supporting GeneA–GeneB > GeneA–GeneC, then GeneA–GeneC is discarded assuming GeneA–GeneB is the correct fusion and the evidence for GeneA–GeneC likely stems from mismapping.
- Promiscuous fusion filter: if candidate GeneA–GeneB exists along with alternative fusion candidates GeneA–GeneC and GeneA–GeneD, and the fusion evidence supporting GeneA–GeneB greatly exceeds that of the alternative fusions (at least 20× support), the alternatives are discarded and the dominant fusion pair is retained. If afterwards, GeneA is found to have at least ten fusion partners, all GeneA-containing fusion pairs are excluded from the sample altogether.
- “Red herring” filter: fusion pairs are annotated using FusionAnnotator [82] with the CTAT Human Fusion Lib database release v0.1.0 [83]. Any fusion pair annotated as having been found in normal RNA-seq data sets, including a mitochondrial or HLA gene partner, is discarded. Any fusion involving gene pairs that are both immunoglobulin gene segments are also discarded.
- Fusion expression filter: the abundance of RNA-seq fragments supporting the fusion are normalized according to sequencing depth as fusion fragments per million total RNA-seq fragments (FFPM). Fusion candidates having less than 1 evidence fragment per 10 M total reads (0.1 FFPM) are discarded as insufficiently supported. The 0.1 FFPM corresponds to the 0.99 quantile of FFPM values for non-recurrent fusions identified in GTEx samples (data not shown).

The advanced fusion filtering described above is implemented in our “FusionFilter” [84] software module shared among CTAT fusion software. STAR-Fusion code and documentation is available on GitHub at [43]. STAR-Fusion was executed from with a Docker image containing all software, including the FusionAnnotator and FusionFilter modules, as provided on DockerHub [85].

### Fusion prediction by TrinityFusion

An overview of the TrinityFusion pipeline is provided as Additional file 2: Figure S13. The TrinityFusion pipeline uses the Trinity assembler to de novo reconstruct transcript sequences from RNA-seq, and GMAP [49, 50] to then align the transcripts to the genome to identify candidate chimeric sequences. The fusion candidates are examined to remove likely assembly artifacts, and read support for the fusion is estimated by leveraging Bowtie2 [29] to align the original RNA-seq reads to the Trinity fusion transcripts to further classify reads as fusion spanning or junction reads.

TrinityFusion has three different execution modes based on the inputs to be used for de novo reconstruction and subsequent fusion detection:

- TrinityFusion-D performs Trinity de novo assembly on all input reads.
- TrinityFusion-C restricts Trinity de novo assembly to only those reads defined as chimeric or discordant according to STAR genome alignments.
- TrinityFusion-UC utilizes both the chimeric and discordant reads along with all reads that fail to align to the genome according to STAR.

For execution modes TrinityFusion-C and TrinityFusion-UC, there is a prerequisite that STAR has been executed (as described above for STAR-Fusion) to generate the genome read alignments (bam output file) and the STAR Chimeric.out.junction that defines the discordant and chimeric read alignments. TrinityFusion uses these reports to define the target reads and then extracts them from the input FASTQ files to create inputs for Trinity de novo assembly.

The reconstructed transcripts are aligned to the human reference genome as provided in the CTAT genome lib (see below) like so, using GMAP:

gmap -D $GMAP_DB_DIR -d $GMAP_DB_NAME Trinity.fasta -f 3 -n 0 -x 30 -t $CPU > gmap.gff3.

The chimeric alignments defined by GMAP are then further annotated according to overlap with reference gene annotations. To avoid likely false positives arising from misassembly of related sequences, we examine the precision of the alignment at the breakpoint between the two gene candidates. Each candidate chimeric transcript assembly sequence is extracted and split with 25 base overhangs at the putative breakpoint. Then, each split sequence with overhang is realigned to the reference genome using GMAP to determine the extent of the alignment into the overhang region at each putative chimeric locus. If alignments extend beyond 12 bases into the overhang region, that candidate fusion transcript is eliminated as a likely assembly artifact between sequence-similar genes. This fuzzy alignment logic was inspired by a similar process performed by JAFFA-assembly [32] that examines fuzzy boundaries of candidate chimeric BLAT [27] alignments.

All input reads are then aligned against the remaining candidate assembled chimeric fusion transcripts using Bowtie2 like so:

bowtie2 -k10 -p 4 --no-mixed --no-discordant --very-fast --end-to-end -x $bowtie2_target -1 $left_fq_file -2 $right_fq_file

Reads spanning or overlapping the fusion breakpoint are counted. The breakpoint is required to precisely match reference exon splice sites, as allowing for non-reference splice junctions was found to greatly inflate the false-positive rate (data not shown). At least two RNA-seq fragments must align across or span the breakpoint supporting the fusion. If there are only breakpoint-overlapping reads and no spanning fragments, then we ensure that the 12 bases on both sides of the breakpoint are of sufficient sequence complexity, requiring an entropy ≥ 1.5.

Finally, fusion gene pairs are filtered according to the same “advanced” filtering criteria leveraged by STAR-Fusion as implemented in the FusionFilter module, considering paralogs, promiscuity, and potential red herrings.

TrinityFusion software organization: TrinityFusion, as other Trinity CTAT software pipelines, is implemented as a set of software modules that can be easily shared among Trinity CTAT applications for flexible execution wherever shared functionality is desirable. The TrinityFusion discordant and unmapped read assembly is encapsulated by a DISCASM module [86]. The assembled transcript chimeric alignment detection is encapsulated by our GMAP-fusion module [87]. Both DISCASM and GMAP-fusion are then leveraged as shared submodules that define the TrinityFusion software. TrinityFusion software code and documentation is available on GitHub at [88]. TrinityFusion was executed as a Singularity image built from the Docker image available at DockerHub [89].

### The CTAT genome library leveraged by STAR-Fusion and TrinityFusion

The CTAT genome library includes the human reference genome, reference gene structure annotations, and a database of all-vs-all blastn alignments among the reference transcript sequences used for paralog detection and evaluating potential read mismappings between similar gene sequences. The CTAT genome library used in this study includes the human hg19 reference genome and GENCODE v19 gene annotations [73]. Blastn alignments were generated separately for reference coding sequences (CDS) supplemented with long noncoding RNAs (lncRNAs) and for reference cDNA sequences (including untranslated regions) as follows:

All-vs-all blastn search using CDS and lncRNAs: “blastn -query ref_annot.cdsplus.fa -db ref_annot.cdsplus.fa -max_target_seqs 10000 -outfmt 6 -evalue 1e-10 -num_threads $CPU -dust no > ref_annot.cdsplus.allvsall.outfmt6”

All-vs-all blastn search using cDNA sequences: “blastn -query ref_annot.cdna.fa -db ref_annot.cdna.fa -max_target_seqs 10000 -outfmt 6 -evalue 1e-10 -num_threads $CPU -dust no > ref_annot.cdna.allvsall.outfmt6”

The above-generated “ref_annot.cdsplus.allvsall.outfmt6” alignments are used for candidate paralog detection, and the above “ref_annot.cdna.allvsall.outfmt6” alignments are used for assessing read alignments between gene pairs. This latter file includes alignments between UTR regions that may confound read mappings but may not indicate evolutionarily relatedness between corresponding genes (i.e., alignments among repeats in UTR regions).

The CTAT genome lib also incorporates our human fusion library [83], which incorporates lists of fusions relevant to cancer, and those identified among normal tissues and unlikely to be relevant to cancer biology. The cancer-relevant fusions include those reported in the Mitelman Database of Chromosome Aberrations and Gene Fusions in Cancer [90], ChimerDB 2.0 [91], COSMIC [92, 93], and fusions discovered in cancer cell lines and surveys of tumor samples [1, 94, 95]. Those fusions found in normal tissues comprise our red herrings list and include those previously identified via our internal screens of GTEx data, our exploration of the Illumina human body map data [75], previous reports of fusions found in normal tissue samples [96–98], and lists of gene families and paralogs that may confound fusion prediction [99, 100]. FusionCatcher uses many of these same resources, and we credit FusionCatcher for inspiring the development of our growing collection and our companion utility FusionAnnotator [82] for annotating gene fusions accordingly. FusionAnnotator comes bundled as a shared software module in both CTAT fusion tools STAR-Fusion and TrinityFusion.

## Supplementary information

**Additional file 1.** **Table S1.** simulated 50 base PE read fusion predictions; **Table S2.** simulated 101 base PE read predictions; **Table S3.** known experimentally validated fusions; **Table S4.** fusion predictions on cancer cell lines; **Table S5.** Centrifuge-based viral and microbial source predictions for Trinity-assembled transcripts; **Table S6.** Viral assignments of Trinity-UC assembled transcripts via blastn; **Table S7.** Fusion prediction software commands; **Table S8.** sources of RNA-seq data for modeling simulated RNA-seq; **Table S9.** Cancer cell lines targeted for fusion discovery.

**Additional file 2:** **Figure S1.** Prediction counts vs. minimum sum evidence fragments for the simulated 50 base length PE reads; **Figure S2.** Prediction counts vs. minimum sum evidence fragments for the simulated 101 base length PE reads; **Figure S3.** TPR and PPV at maximum F1 for fusion predictions based on simulated data sets; **Figure S4.** Contrasting de novo transcript reconstructions according to TrinityFusion execution mode; **Figure S5.** TPR vs. PPV at maximum F1 for each truth set; **Figure S6.** Adjustments in counts of TP and FP after accepting likely paralogs as proxies for known fusion partners; **Figure S7.** Correlation of fusion predictions among methods on the cancer cell line data sets; **Figure S8.** Impact of ‘allow paralogs’ and ‘ignore uncertain’ fusions on accuracy rankings; **Figure S9.** Distribution of accuracy rankings with the paralog proxy allowance disabled; **Figure S10.** Overview of the STAR-Fusion pipeline; **Figure S11.** Filtering of chimeric and discordant reads; **Figure S12.** Basic criteria for filtering preliminary fusion candidates; **Figure S13.** TrinityFusion Pipeline.

**Additional file 3.** Review history.

## Data Availability

All software and data are publicly and freely available. The software and documentation is made available through GitHub, including software packages STAR-Fusion https://github.com/STAR-Fusion/STAR-Fusion/wiki [43], TrinityFusion https://github.com/trinityrnaseq/TrinityFusion/wiki [47], and our fusion simulation https://github.com/FusionSimulatorToolkit/FusionSimulatorToolkit/wiki [72] and benchmarking code https://github.com/fusiontranscripts/FusionBenchmarking/wiki [71]. Our benchmarking data are made publicly available from our Broad data repository, including the samples with simulated 50 or 101 base PE reads https://data.broadinstitute.org/Trinity/CTAT_FUSIONTRANS_BENCHMARKING/on_simulated_data/ [77], and samples corresponding to the 60 cancer cell lines https://data.broadinstitute.org/Trinity/CTAT_FUSIONTRANS_BENCHMARKING/on_cancer_cell_lines/ [78].
