# Supplementary material for: Accuracy assessment of fusion transcript detection via read-mapping and de novo fusion transcript assembly-based methods
Source: Genome Biol. 2019 Oct 21;20:213. doi: 10.1186/s13059-019-1842-9 (PMC6802306; doi:10.1186/s13059-019-1842-9)
Supplement: Supplementary file 2 — Additional file 2: Figure S1. Prediction counts vs. minimum sum evidence fragments for the simulated 50 base length PE reads; Figure S2. Prediction counts vs. minimum sum evidence fragments for the simulated 101 base length PE reads; Figure S3. TPR and PPV at maximum F1 for fusion predictions based on simulated data sets; Figure S4. Contrasting de novo transcript reconstructions according to TrinityFusion execution mode; Figure S5. TPR vs. PPV at maximum F1 for each truth set; Figure S6. Adjustments in counts of TP and FP after accepting likely paralogs as proxies for known fusion partners; Figure S7. Correlation of fusion predictions among methods on the cancer cell line data sets; Figure S8. Impact of ‘allow paralogs’ and ‘ignore uncertain’ fusions on accuracy rankings; Figure S9. Distribution of accuracy rankings with the paralog proxy allowance disabled; Figure S10. Overview of the STAR-Fusion pipeline; Figure S11. Filtering of chimeric and discordant reads; Figure S12. Basic criteria for filtering preliminary fusion candidates; Figure S13. TrinityFusion Pipeline. [file 13059_2019_1842_MOESM2_ESM.pdf]

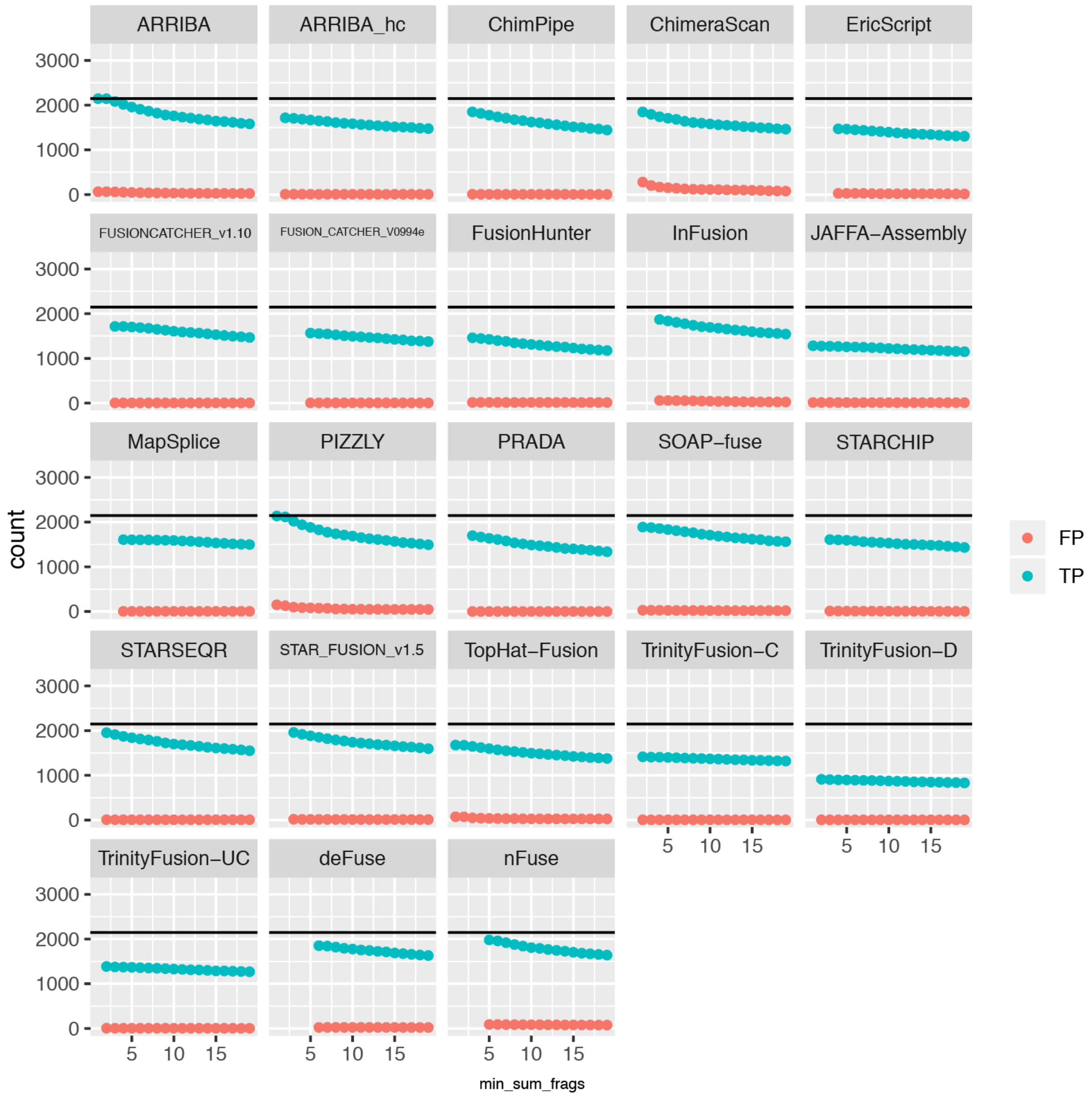

Figure S1: Prediction counts vs. minimum sum evidence fragments for the simulated 50 base length PE reads. True and false positive fusion prediction counts are shown as a function of minimum total evidence fragments required. A black line is drawn at the position of maximum sensitivity for any method.

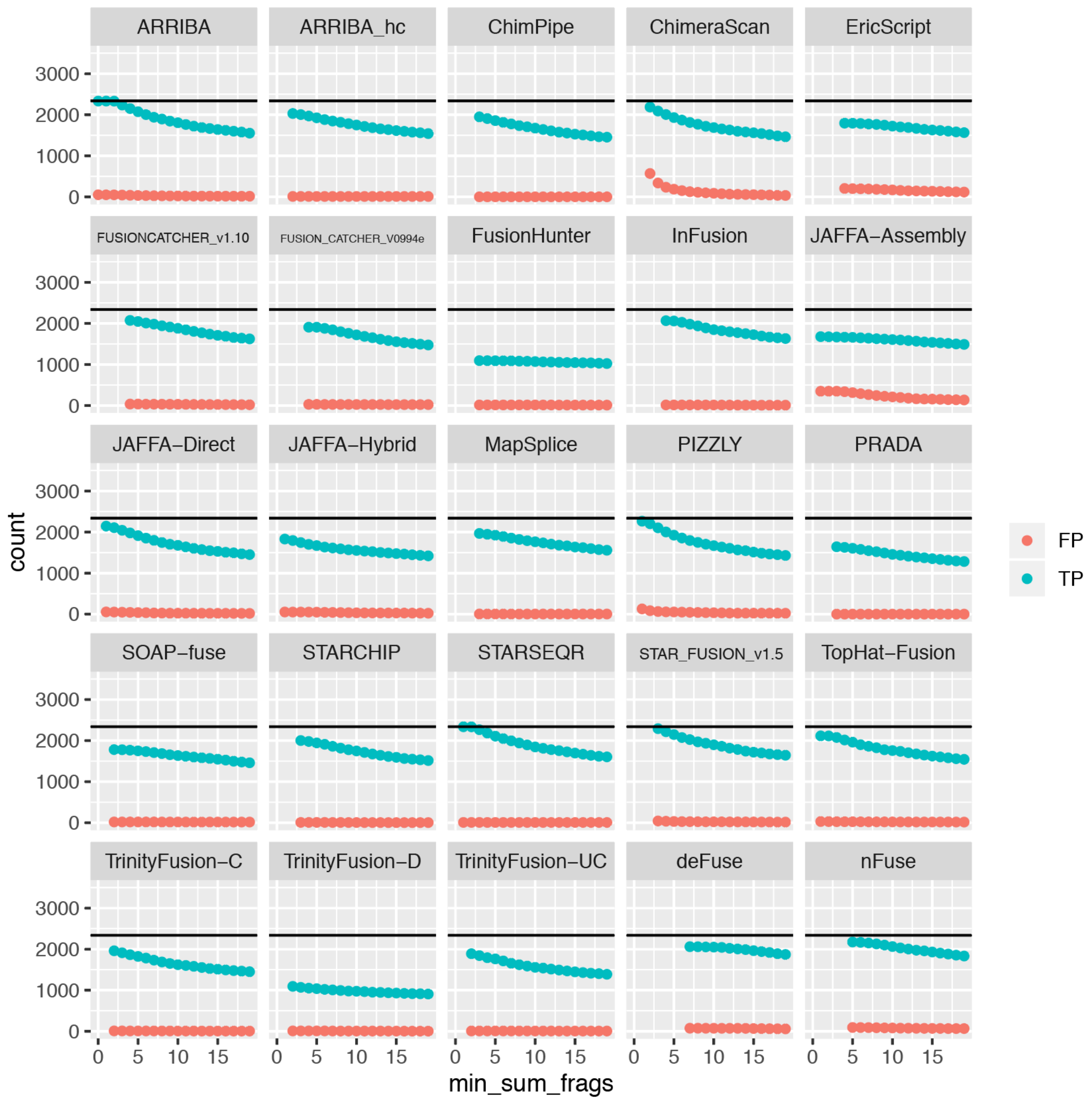

Figure S2: Prediction counts vs. minimum sum evidence fragments for the simulated 101 base length PE reads. True and false positive fusion prediction counts are shown as a function of minimum total evidence fragments required. A black line is drawn at the position of maximum sensitivity for any method.

A.

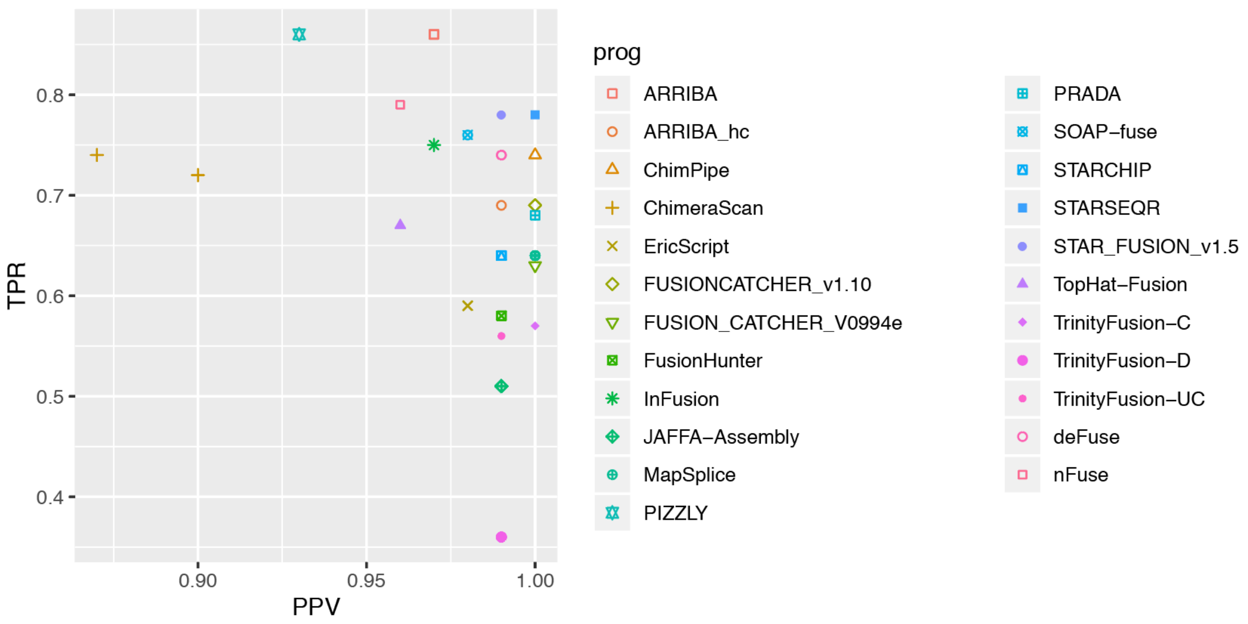

B.

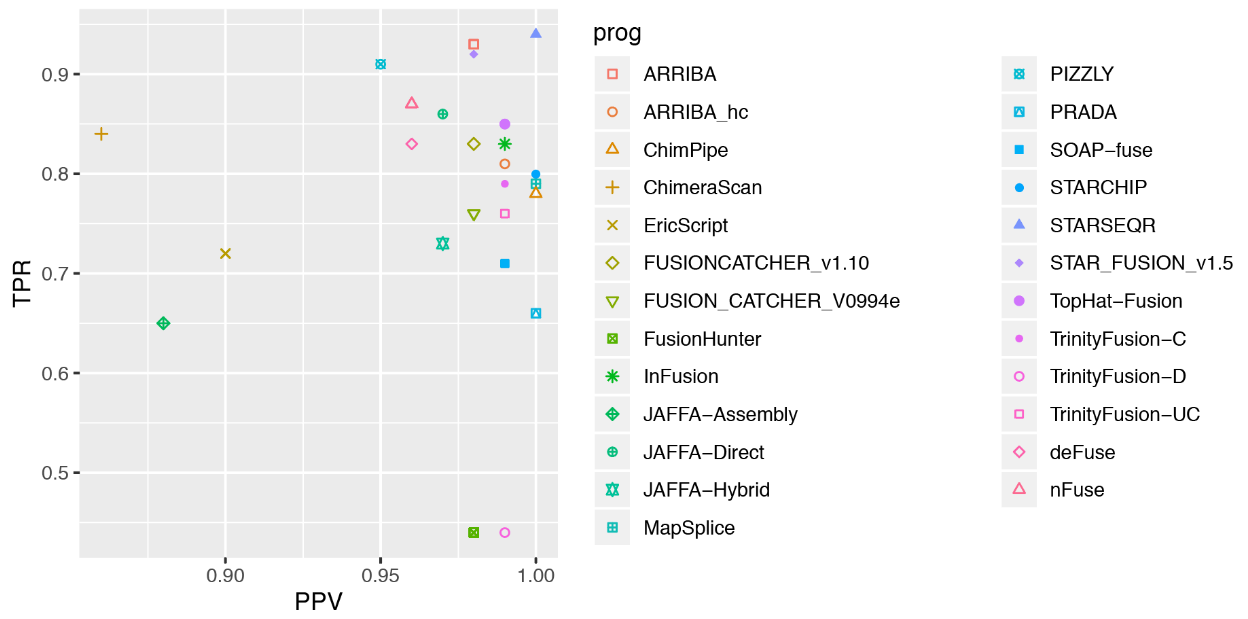

Figure S3: TPR and PPV at maximum F1 for fusion predictions based on simulated data sets (A) 50 base length PE and (B) 101 base length PE reads. JAFFA-Direct and JAFFA-Hybrid were not compatible with the 50 base length reads and so have values shown for only the 101 base length read set. ChimeraScan accumulates substantial numbers of false positive predictions on each data set. Methods are shown with multiple data points whenever there were ties for maximum F1 scores based on the PPV and TPR values at different minimum evidence thresholds applied.

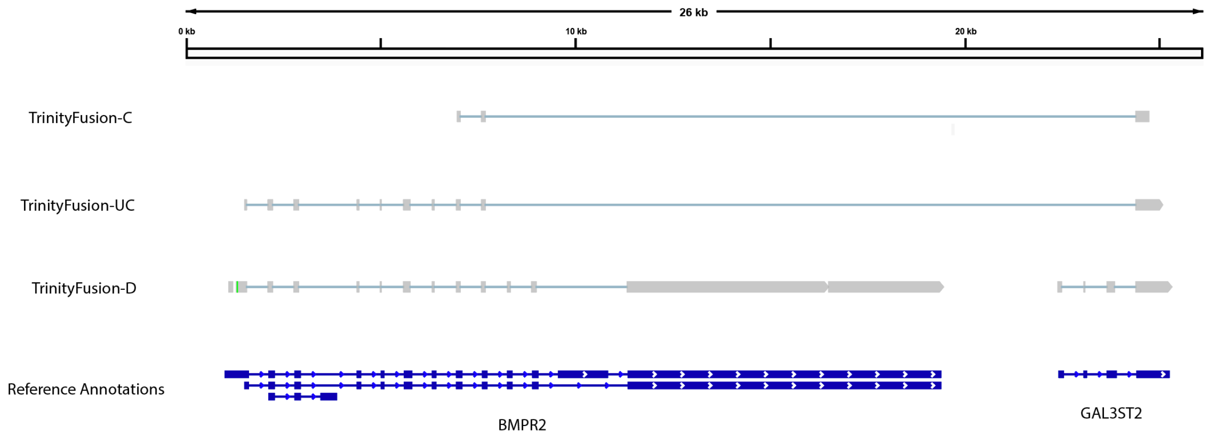

Figure S4: Contrasting de novo transcript reconstructions according to TrinityFusion execution mode. As shown in this example, TrinityFusion-D often reconstructs the normal (unfused) transcripts from all the RNA-seq reads, in contrast to TrinityFusion-C and TrinityFusion-UC, which preferentially *de novo* reconstruct the fusion transcript from chimeric or combination of chimeric and unmapped reads, respectively. Reference annotations for BMPR2 and GAL3ST2 genes are shown at bottom and positioned adjacent in a fabricated single genomic contig to which Trinity-reconstructed transcripts were aligned using GMAP. Only relevant alignments are shown above for clarity.

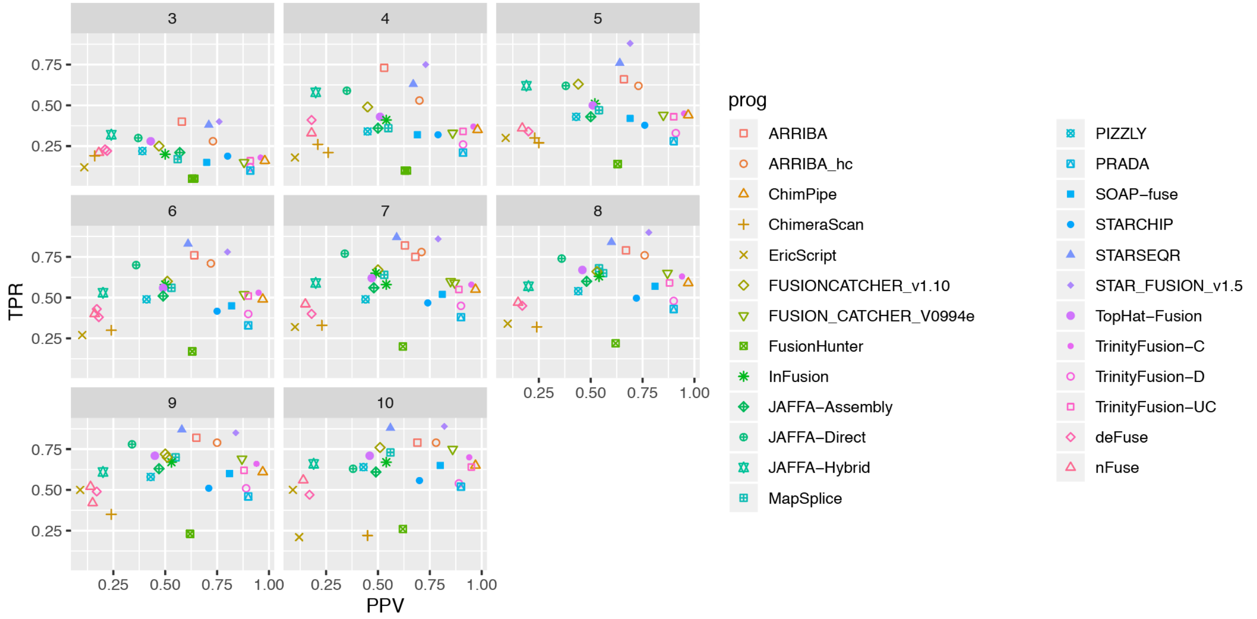

Figure S5: TPR vs. PPV at maximum F1 for each truth set. Each truth set was defined as requiring at least *n* methods agree on the fusion prediction, with *n* set between 3 and 10. The high sensitivity and high specificity groupings are most evident for n between 4 and 8. Beyond 8, the truth sets greatly shrink in size and the groupings begin to coalesce.

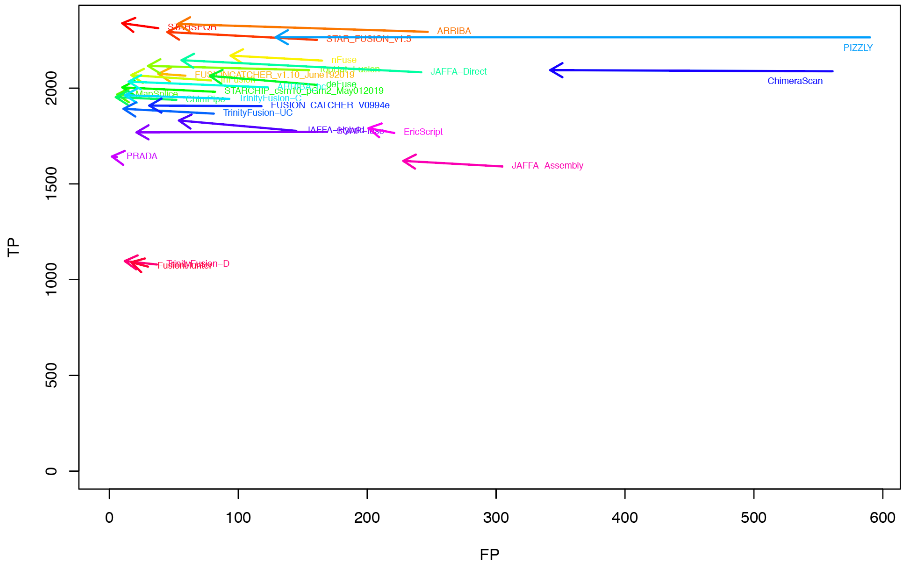

Fig S6: Adjustments in counts of TP and FP after accepting likely paralogs as proxies for known fusion partners. The adjustment mostly reduces the number of perceived FP while only slightly elevating TPs. Results shown here correspond to the simulated 101 base length PE benchmarking data set.

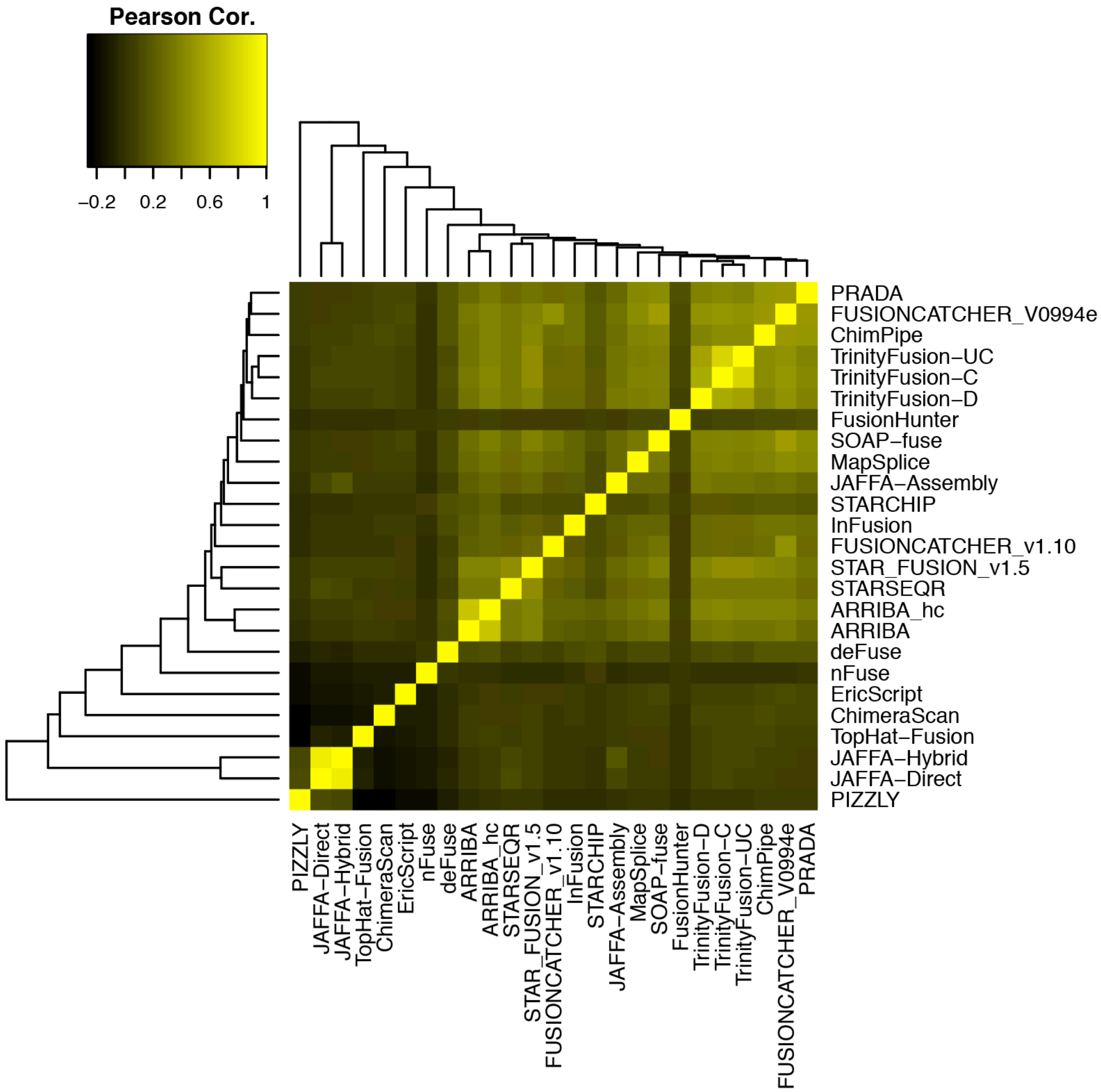

Figure S7: Correlation of fusion predictions among methods on the cancer cell line data sets. JAFFA-Hybrid and JAFFA-Direct are highly correlated, and so JAFFA-Hybrid was excluded from contributing votes towards defining truth sets according to the 'wisdom of crowds' approach. Similarly, only TrinityFusion-C of the TrinityFusion variable execution modes was allowed to contribute to the voting scheme. Arriba_hc was evaluated separately from Arriba but did not contribute votes independently from Arriba.

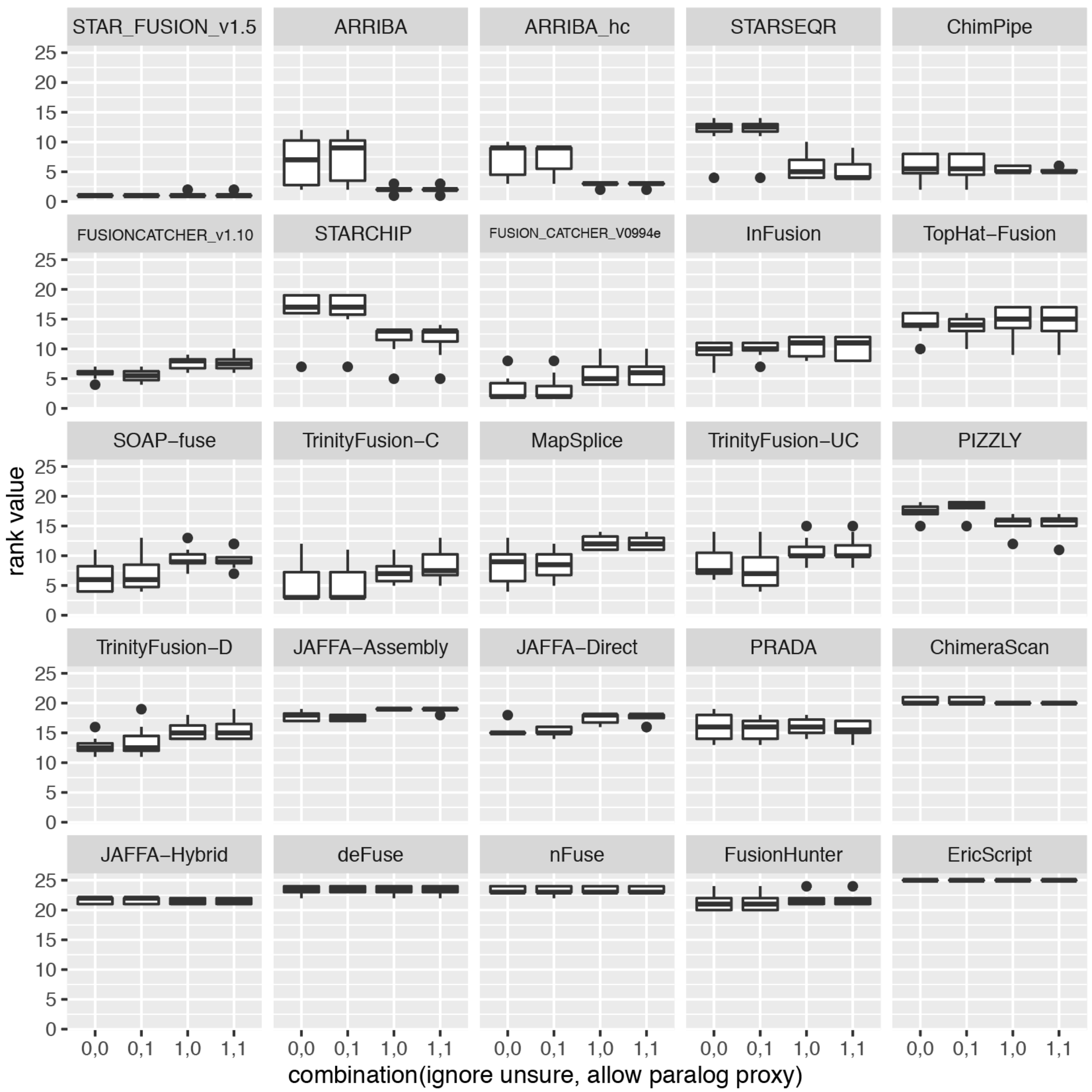

Figure S8: Impact of 'allow paralogs' and 'ignore uncertain' fusions on accuracy rankings. 0=off, 1=on. The paralog proxy allowance had less of an impact on relative rankings than ignoring the unsure fusions (ie. those predictions predicted by *x* methods where $1 < x < n$).

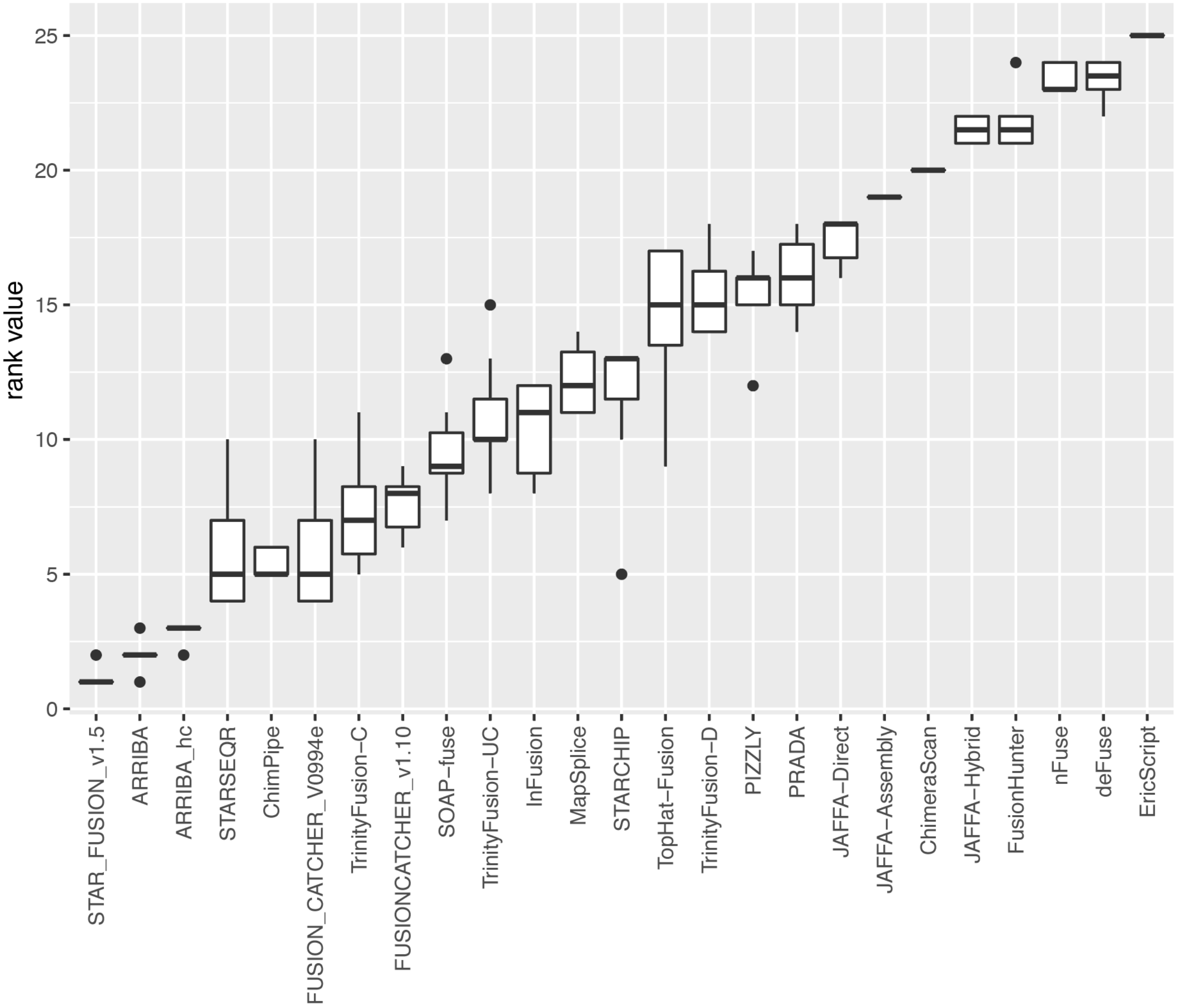

Figure S9: Distribution of accuracy rankings with the paralog proxy allowance disabled. Overall, there is little effect on the distributions of rankings after disabling the paralog proxy allowance, and the top-ranking methods remain unchanged.

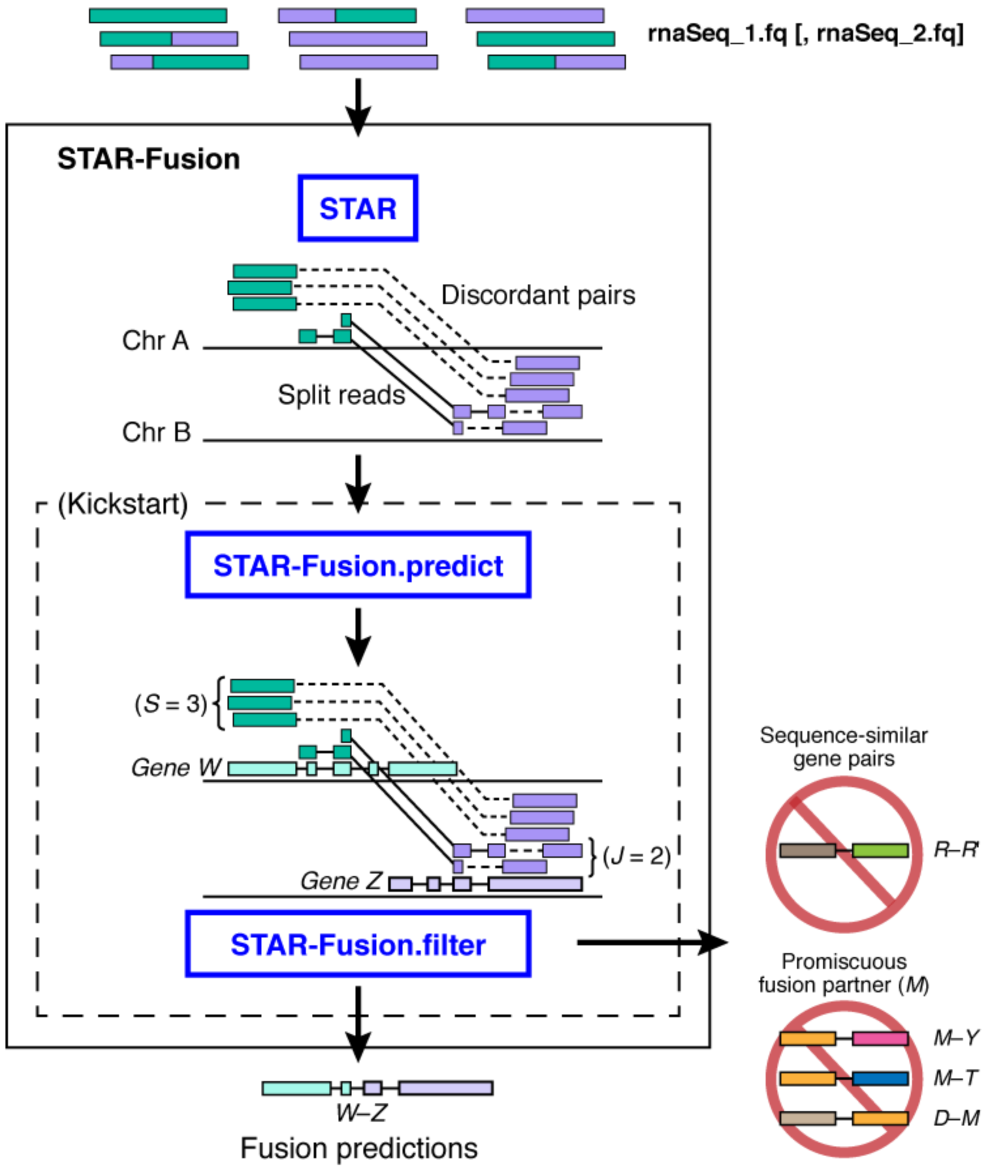

Figure S10: Overview of the STAR-Fusion pipeline. Illumina RNA-Seq reads are aligned to the genome using STAR. Discordant and split-read alignments are identified, mapped to reference transcript structure annotations, filtered to remove likely artifacts, and scored according to the abundance of fusion-supporting reads. Fusion candidates containing sequence-similar gene pairs or promiscuous fusion partners are excluded as likely false positives.

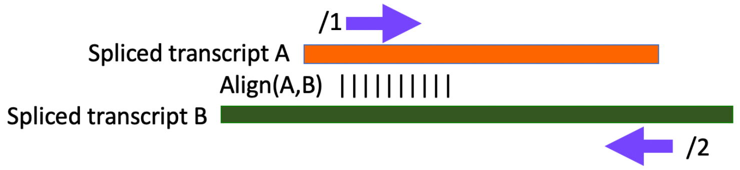

Fig S11: Filtering of chimeric and discordant reads. Reads found to overlap in regions that have detected sequence similarity between the candidate target genes are discarded.

A.

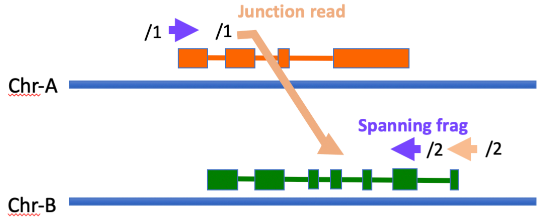

B.

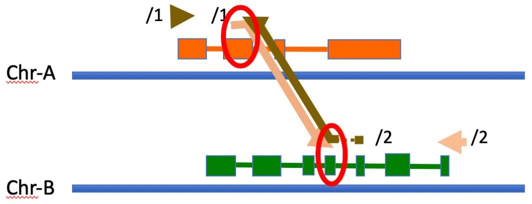

Figure S12: Basic criteria for filtering preliminary fusion candidates. A. At least two fusion supporting RNA-seq fragments are required, and one must be a split read that defines the junction breakpoint. B. In the case there are no spanning fragments and only split/junction reads, we require that there be at least 25 base length of alignment on each side of the fusion transcript breakpoint (red circles).

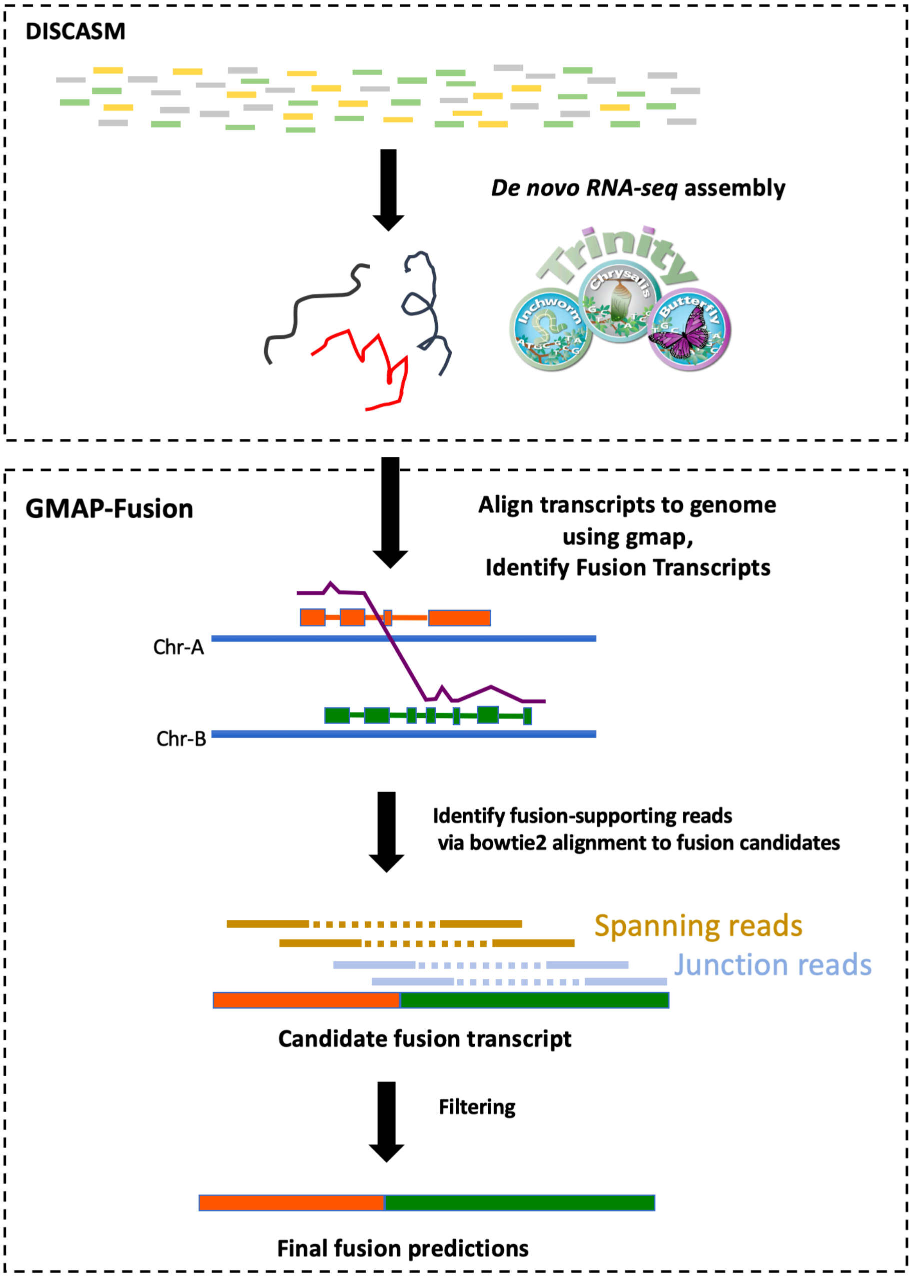

Figure S13: TrinityFusion Pipeline. TrinityFusion consists of two software modules, DISCASM and GMAP-fusion. DISCASM extracts discordant and unmapped reads based on STAR alignment results and runs Trinity to de novo assemble transcripts from these input reads. GMAP-fusion is then run to identify candidate fusion transcripts that yield chimeric alignments to the reference genome. Reads are aligned to the candidate fusion transcripts using bowtie2, followed by filtering according to fusion evidence abundance and more advanced criteria as described in the Methods.
