# Supplementary material for: Accuracy assessment of fusion transcript detection via read-mapping and de novo fusion transcript assembly-based methods
Source: Genome Biol. 2019 Oct 21;20:213. doi: 10.1186/s13059-019-1842-9 (PMC6802306; doi:10.1186/s13059-019-1842-9)
Supplement: Supplementary file 3 — Additional file 3. Review history. [file 13059_2019_1842_MOESM3_ESM.docx]

Review History

**First round of review**

**Reviewer 1**

**Are you able to assess all statistics in the manuscript, including the appropriateness of statistical tests used?**

Yes: The authors report simple statistics and there doesn't seem to be any issue with them, nor a need for other statistics.

**Comments to author:**

This is a benchmarking study where the authors study the performance (accuracy and running time) of 23 methods for fusion transcript detection. The study is straightforward and the findings and recommendations about methods are very valuable to the user community in this field. The authors assess the performance of the methods on both simulated data and biological data. For the biological data, the authors use consensus results from the methods to designate the ground truth. 
The study is very well described. In particular, the authors describe their new methods STAR-Fusion and TrinityFusion in detail. The experiments and metrics are well described. The documentation on ftp and GitHub are very clear. The tables are great, especially with the details of how they ran all the methods. 

* In the simulation setup (the way the data was generated), I didn't see any information on simulating error in the read data. Is it reasonable to assume the reads have no error in them? There's a paragraph in the Discussion section on not modeling some characteristics of the data. But I'm asking here about a simpler characteristic: In real data sets, aren't there usually errors in the reads that would affect the quality of the assembly (whether mapping-based or de novo)? For example, is it realistic to expect almost 0 false positives on real data in this field? Could the lack of simulating error in the data explain why the synthetic data sets are "easy" for all 23 methods when focusing on false positives? 
* Is the finding that TrinityFusion-D is outperformed by TrinityFusion-C and TrinityFusion-UC intuitive to the authors? I find it a bit surprising because the -D version uses all the reads, which I would have expected to give more signal or power to detection. 
* The last paragraph in the Discussion section talks about the need to improve existing methods. The current study falls more on the descriptive side than the analytical one. Can the authors elaborate a bit more on where they see room or directions for improving existing methods? 
* The authors reference four papers that seem to review methods for the same task, namely references 10, 11, 15, and 16, and some of these also report on accuracy using synthetic data sets (e.g., reference 15). Can the authors please discuss what their study adds to these existing ones? It definitely seems like the current study is much more comprehensive in terms of the methods assessed. But, how do the findings here compare to those reported before on the methods that are shared between the studies? 
* A minor point: On page 8, the authors write "Of 45 experimentally validated ..., and 84 were predicted by at least 3 methods." There is something strange here as 84 out of 45 doesn't make sense. Further confusion comes from trying to read Fig. 3(a). I was expecting 45 fusion events (45 columns), but clearly there are more. The caption doesn't explain this. Please clarify Fig. 3 and the statement about how many were predicted by at least 3 methods. 
* Related to the very last comment, the manuscript would benefit from a more careful proofreading. There are awkward phrases and and grammatical issues. For example, maybe it's better to use "real data" or "empirical data" than "genuine data." As another example, the very last sentence in the Abstract sounds disconnected from the rest of the Abstract. I believe the authors wanted to say that the lower accuracy of de novo assembly-based methods notwithstanding, these methods would be useful for reconstructing fusion isoforms and tumor viruses, both of which are important in cancer research. The adverse "Conversely" in the second paragraph of Background doesn't seem to be appropriate as the statement about RNA-seq doesn't negate or contrast anything in the preceding statement. 

Best regards,
Luay Nakhleh 
(I sign all my reviews)

**Reviewer 2**

**Are you able to assess all statistics in the manuscript, including the appropriateness of statistical tests used?**

Yes

**Comments to author:**

Predicting gene fusion events is important for cancer diagnosis and therapeutic applications. Therefore, it is a task of great biological and clinical importance to address what the most suitable data and the high-performance tools for fusion detection are. The authors evaluated a total of 23 methods using different RNA-seq data sets (including two developed by their group) and recommend a few methods that were generally better than others in identifying fusions from cancer RNA-seq data. However, there are still several issues and questions to be addressed.

1.     Each method might have a preferred read aligner to achieve a higher accuracy.  For read-mapping based methods, did the authors use the same aligner? Or did you use each algorithm's preferred aligner if they do have one? For example, did SOAP-fuse work better with SOAP2 (the same tool developed by the same group)? A summary table that presents the algorithm's preferred aligner would be helpful.
2.     The 23 methods were compared using both simulated data and real cancer RNA-seq data under different expression levels and read lengths. But several other important factors, for example, insert size, were not considered.  
3.     The median run time on cancer cell lines was used to rank the methods. But does the run time correlate with the number of potential fusions, or is run time sensitive to coverage? A computational cost comparison is expected.
4.     The section "Exploration of de novo reconstructed transcripts of potential foreign origin" considered only TrinityFusion while other de novo assembly based methods, such as JAFFA and JAFFA-Hybrid were not included. While experimentally validated fusions with bacterial/virus genes were not available, it might be helpful to use some simulated data sets to do the comparison.
5.     Please use consistent font styles for method names. (For example, Figure 4a and Figure 4b)

**Authors Response**

**Point-by-point responses to the reviewers’ comments:**

\# Response to Reviewer 1: 

* This is a benchmarking study where the authors study the performance (accuracy and running time) of 23 methods for fusion transcript detection. The study is straightforward and the findings and recommendations about methods are very valuable to the user community in this field. The authors assess the performance of the methods on both simulated data and biological data. For the biological data, the authors use consensus results from the methods to designate the ground truth. The study is very well described. In particular, the authors describe their new methods STAR-Fusion and TrinityFusion in detail. The experiments and metrics are well described. The documentation on ftp and GitHub are very clear. The tables are great, especially with the details of how they ran all the methods. 

*We thank the Reviewer for their appreciation of our work.* 

* In the simulation setup (the way the data was generated), I didn't see any information on simulating error in the read data. Is it reasonable to assume the reads have no error in them? There's a paragraph in the Discussion section on not modeling some characteristics of the data. But I'm asking here about a simpler characteristic: In real data sets, aren't there usually errors in the reads that would affect the quality of the assembly (whether mapping-based or de novo)? For example, is it realistic to expect almost 0 false positives on real data in this field? Could the lack of simulating error in the data explain why the synthetic data sets are "easy" for all 23 methods when focusing on false positives? 
 *We thank the Reviewer for this important question. The simulated RNA-seq data take into account error profiles and fragment length distributions among the read sequences, as these are modeled by RSEM based on real RNA-seq data. However, the simulations do not sufficiently account for other types of artifacts, such as reverse transcription template switching that could result in false fusion detections with real data. We clarify this in the revised methods and discussion. 

In the Discussion (p. 14), we now indicate as a remaining opportunity “…, developing more realistic RNA-seq simulations, …”. In the Methods (p. 16), we now state “Note, however, that while the read sequence simulations model sequence and fragment length characteristics of real RNA-seq data, the current simulations do not model reverse transcription template switching or other important confounding characteristics of real RNA-seq data that are relevant to fusion detection. “ 

All methods did encounter false positives with the simulated data sets, but the numbers of false positives were one to two orders of magnitude fewer than the numbers of true positives and so were largely negligible in their contribution to overall accuracy estimates. We now elaborate on this on page 7, stating “On the simulated data, accuracy was almost entirely a function of sensitivity for fusion detection, as most methods exhibited few false positives (1-2 orders of magnitude lower).”* 

* Is the finding that TrinityFusion-D is outperformed by TrinityFusion-C and TrinityFusion-UC intuitive to the authors? I find it a bit surprising because the -D version uses all the reads, which I would have expected to give more signal or power to detection. 

*Since TrinityFusion-D was not specifically targeted to chimeric and unmapped reads, it would often reconstruct the normal (unfused) versions of transcripts preferentially rather than in addition to the chimeric transcripts, and this likely depends on the relative expression of fusion transcripts compared to the unfused counterparts. We now elaborate on this in the manuscript (p. 8) and include a new Supplementary Figure 4 as an illustrative example.* 

* The last paragraph in the Discussion section talks about the need to improve existing methods. The current study falls more on the descriptive side than the analytical one. Can the authors elaborate a bit more on where they see room or directions for improving existing methods? 

*We thank the Reviewer for this important suggestion. We now expand on this section of the Discussion (p.14) to include "... there remain opportunities for improving fusion transcript prediction accuracy, developing more realistic RNA-seq simulations, and expanding the catalog of experimentally validated fusion transcripts.". 

In our future work, we will identify areas of improvement for the fusion tools that we have been developing, including STAR-Fusion and TrinityFusion. We have developed a new software tool, FusionInspector, and are applying it to explore those cases where our tools have failed, to identify fusions that were identified by other methods. This should provide clues towards yielding further algorithmic improvements.* 

* The authors reference four papers that seem to review methods for the same task, namely references 10, 11, 15, and 16, and some of these also report on accuracy using synthetic data sets (e.g., reference 15). Can the authors please discuss what their study adds to these existing ones? It definitely seems like the current study is much more comprehensive in terms of the methods assessed. But, how do the findings here compare to those reported before on the methods that are shared between the studies? 

*We thank the Reviewer for this important suggestion. We now directly address this in the Introduction (p.4) and Discussion (p. 12). 

In the Introduction we explain the limitations of previous studies: "Earlier evaluations of RNA-seq based fusion prediction methods have highlighted shortcomings of contemporary methods, but were mostly limited to small numbers of fusion candidates, and relied heavily on simulated test data for accuracy assessment (15, 16). Here, we advance fusion transcript prediction benchmarking to include thousands of fusion transcripts at varied expression levels, and devise a strategy to benchmark fusion accuracy leveraging real cancer transcriptome data without a priori defined gold standard truth sets." 

In the Discussion, we mention specific differences in findings: "In contrast to an earlier assessment of fusion prediction accuracy that found EricScript to be top-ranking (16), our assessment on cancer cell line RNA-seq positions EricScript as least accurate among those examined, suffering mostly from a high rate of false positives."* 

* A minor point: On page 8, the authors write "Of 45 experimentally validated ..., and 84 were predicted by at least 3 methods." There is something strange here as 84 out of 45 doesn't make sense. Further confusion comes from trying to read Fig. 3(a). I was expecting 45 fusion events (45 columns), but clearly there are more. The caption doesn't explain this. Please clarify Fig. 3 and the statement about how many were predicted by at least 3 methods. 

*Thank you for pointing this out this confusing phrasing. In our revised text (p. 8-9), we have clarified it as follows: "Of a total of 86 fusions predicted by at least three methods, we find 44 experimentally validated fusions (Figure 3a).". Moreover, we have reorganized most of that paragraph for improved clarity and to reflect changes resulting from our updated analysis with the most current version of the FusionCatcher software.* 

* Related to the very last comment, the manuscript would benefit from a more careful proofreading. There are awkward phrases and and grammatical issues. For example, maybe it's better to use "real data" or "empirical data" than "genuine data." 

*We appreciate the reviewer's sentiment and have carefully proofread the manuscript. All text updates are easily identified in the 'tracked changes' version of the manuscript. We agree that 'real' is a better word choice than 'genuine' in the context of RNA-seq data and have replaced all such occurrences.* 

* As another example, the very last sentence in the Abstract sounds disconnected from the rest of the Abstract. I believe the authors wanted to say that the lower accuracy of de novo assembly-based methods notwithstanding, these methods would be useful for reconstructing fusion isoforms and tumor viruses, both of which are important in cancer research. 

*We thank the reviewer for this suggested text and have incorporated it into the manuscript verbatim.* 

* The adverse "Conversely" in the second paragraph of Background doesn't seem to be appropriate as the statement about RNA-seq doesn't negate or contrast anything in the preceding statement. 

*Our use of 'Conversely' is meant to relay 'in contrast to whole genome sequencing'. For improved clarity, we updated the text accordingly.* 

* Best regards, Luay Nakhleh (I sign all my reviews) 

*We deeply appreciate the signed review.* 

\# Response to Reviewer 2 

* Predicting gene fusion events is important for cancer diagnosis and therapeutic applications. Therefore, it is a task of great biological and clinical importance to address what the most suitable data and the high-performance tools for fusion detection are. The authors evaluated a total of 23 methods using different RNA-seq data sets (including two developed by their group) and recommend a few methods that were generally better than others in identifying fusions from cancer RNA-seq data. However, there are still several issues and questions to be addressed. 

*We are grateful to the Reviewer for their appreciation of our study.* 

1. Each method might have a preferred read aligner to achieve a higher accuracy. For read-mapping based methods, did the authors use the same aligner? Or did you use each algorithm's preferred aligner if they do have one? For example, did SOAP-fuse work better with SOAP2 (the same tool developed by the same group)? A summary table that presents the algorithm's preferred aligner would be helpful. 

*We thank the Reviewer for this important question. Each method does generally include a preferred aligner or collection of aligners. In Table 1, we identify the aligner used with each method. The software tools are generally tied to specific aligners, and so you cannot easily swap out one alignment tool for another (e.g., swap bowtie2 with STAR). We followed each group’s installation instructions and used specified software versions where indicated. When the developers provided Docker or Singularity images, we used corresponding images with pre-packaged software rather than performing direct installations. We now emphasize this in the revised text (p. 5).* 

2. The 23 methods were compared using both simulated data and real cancer RNA-seq data under different expression levels and read lengths. But several other important factors, for example, insert size, were not considered. 

*We explored fusion prediction accuracy based on conventional RNA-seq and so focused primarily on data derived from standard RNA-seq on cancer cell lines. Our simulated RNA-seq data are also modeled based on conventional RNA-seq data. We agree that it would be useful to further explore alternative RNA-seq library preparations and their impact on fusion detection, including different insert lengths and exploration of long reads from additional sequencing technologies, but we considered this beyond the scope of our current effort. We now state in the revised Discussion (p. 14), “As the sequencing technologies and experimental protocols continue to evolve, the universe of available methods and software will surely continue to expand. Our fusion transcript benchmarking framework provides a flexible system for evaluating these newly developed methods as they become available.”.* 

3. The median run time on cancer cell lines was used to rank the methods. But does the run time correlate with the number of potential fusions, or is run time sensitive to coverage? A computational cost comparison is expected. 

*We thank the Reviewer for this important question. As we show (Figure 4b), the distribution of execution times that we observed nicely differentiates faster from slower methods, and execution times depended more on the software used than the specific sample processed. Note that all samples were limited to 30M PE reads and larger data sets would have led to longer execution times, as runtimes for each program tend to increase as a function of the number of input reads. In the case of STAR-Fusion, even with a sample that contains few to no true fusions, there are still hundreds to thousands of fusion candidates that are explored during the execution and it is only the final filtering stage that yields the smaller set of reported fusions. The number of initial fusion candidates can be related to the number of input reads and other characteristics of the reads (quality, length, etc.), but execution times are not likely to be correlated to the number of final fusions that are reported by many of the tools. Our manuscript focuses mostly on accuracy assessment, and only explores execution time as a way of differentiating faster from slower programs.* 

4. The section "Exploration of de novo reconstructed transcripts of potential foreign origin" considered only TrinityFusion while other de novo assembly based methods, such as JAFFA and JAFFA-Hybrid were not included. While experimentally validated fusions with bacterial/virus genes were not available, it might be helpful to use some simulated data sets to do the comparison. 

*While we did not explore the de novo assembled transcripts generated by JAFFA or JAFFA-Hybrid, we would expect the results of those assemblies to also be useful in this context. Our focus on TrinityFusion for this section was to differentiate among the characteristics of our different TrinityFusion execution modes (UC, C, and D), as each had different fusion-finding capabilities and efficacy for reconstructing foreign transcripts as an auxillary data type. We now specifically mention JAFFA in the manuscript as a potential source of such transcripts (p. 10): “In addition to de novo reconstruction for fusion transcript identification, TrinityFusion, JAFFA, and other de novo assembly-based methods allow us to explore other transcripts that are not well represented by the reference genome sequence or that are lacking from the reference altogether.”* 

5. Please use consistent font styles for method names. (For example, Figure 4a and Figure 4b) 

*We thank the Reviewer for pointing this out. We have carefully checked all figures now for consistent font usage.*
